# Safety and efficacy of a novel anti-CD19 chimeric antigen receptor T cell product targeting a membrane-proximal domain of CD19 with fast on- and off-rates against non-Hodgkin lymphoma: a first-in-human study

**DOI:** 10.1186/s12943-023-01886-9

**Published:** 2023-12-09

**Authors:** Yunlin Zhang, Ruchi P. Patel, Ki Hyun Kim, Hyungwoo Cho, Jae-Cheol Jo, Seong Hyun Jeong, Sung Yong Oh, Yoon Seok Choi, Sung Hyun Kim, Ji Hyun Lee, Mathew Angelos, Puneeth Guruprasad, Ivan Cohen, Ositadimma Ugwuanyi, Yong Gu Lee, Raymone Pajarillo, Jong Hyun Cho, Alberto Carturan, Luca Paruzzo, Guido Ghilardi, Michael Wang, Soohwan Kim, Sung-Min Kim, Hyun-Jong Lee, Ji-Ho Park, Leiguang Cui, Tae Bum Lee, In-Sik Hwang, Young-Ha Lee, Yong-Jun Lee, Patrizia Porazzi, Dongfang Liu, Yoon Lee, Jong-Hoon Kim, Jong-Seo Lee, Dok Hyun Yoon, Junho Chung, Marco Ruella

**Affiliations:** 1 Center for Cellular Immunotherapies, Perelman School of Medicine at the University of Pennsylvania, 3400 Civic Center Boulevard, Perelman Center for Advanced Medicine, SPE 8-112, Philadelphia, PA 19104 USA; 2 Division of Hematology-Oncology, Hospital of the University of Pennsylvania, Philadelphia, PA USA; 3 Biopharmaceutical Research Center, AbClon Inc., #1401, Ace Twin Tower1, 285 Digital-Ro, Guro-Gu, Seoul, Korea; 4 Department of Oncology, Asan Medical Center, University of Ulsan College of Medicine, 88 Olympic-Ro 43-Gil, Songpa-Gu, Seoul, Korea; 5 Ulsan University Hospital, University of Ulsan College of Medicine, Ulsan, Korea; 6 Ajou University Hospital, Suwon, Korea; 7 Division of Hematology-Oncology, Department of Internal Medicine, Dong-A University College of Medicine, Busan, Korea; 8 College of Pharmacy and Institute of Pharmaceutical Science and Technology, Hanyang University, Ansan, Korea; 9 Department of Pathology, Immunology and Laboratory Medicine, Rutgers New Jersey Medical School, Newark, NJ USA; 10 Cancer Research Institute, Seoul National University College of Medicine, Suite 510, Samsung Cancer Research Building, 103 Daehak-Ro, Jongno-Gu, Seoul, Korea; 11 Lymphoma Program, Abramson Cancer Center, University of Pennsylvania, Philadelphia, PA USA

**Keywords:** CD19, CAR T cells, Resistance, CD19 mutations, Epitope masking, Lymphoma, Leukemia, Low avidity, Fast on- and off-rate, Membrane-proximal epitope

## Abstract

**Background:**

Commercial anti-CD19 chimeric antigen receptor T-cell therapies (CART19) are efficacious against advanced B-cell non-Hodgkin lymphoma (NHL); however, most patients ultimately relapse. Several mechanisms contribute to this failure, including CD19-negative escape and CAR T dysfunction. All four commercial CART19 products utilize the FMC63 single-chain variable fragment (scFv) specific to a CD19 membrane-distal epitope and characterized by slow association (on) and dissociation (off) rates. We hypothesized that a novel anti-CD19 scFv that engages an alternative CD19 membrane-proximal epitope independent of FMC63 and that is characterized by faster on- and off-rates could mitigate CART19 failure and improve clinical efficacy.

**Methods:**

We developed an autologous CART19 product with 4-1BB co-stimulation using a novel humanized chicken antibody (h1218). This antibody is specific to a membrane-proximal CD19 epitope and harbors faster on/off rates compared to FMC63. We tested h1218-CART19 in vitro and in vivo using FMC63-CART19-resistant models. We conducted a first-in-human multi-center phase I clinical trial to test AT101 (clinical-grade h1218-CART19) in patients with relapsed or refractory (r/r) NHL.

**Results:**

Preclinically, h1218- but not FMC63-CART19 were able to effectively eradicate lymphomas expressing CD19 point mutations (L174V and R163L) or co-expressing FMC63-CAR19 as found in patients relapsing after FMC63-CART19. Furthermore, h1218-CART19 exhibited enhanced killing of B-cell malignancies in vitro and in vivo compared with FMC63-CART19. Mechanistically, we found that h1218-CART19 had reduced activation-induced cell death (AICD) and enhanced expansion compared to FMC63-CART19 owing to faster on- and off-rates. Based on these preclinical results, we performed a phase I dose-escalation trial, testing three dose levels (DL) of AT101 (the GMP version of h1218) using a 3 + 3 design. In 12 treated patients (7 DLBCL, 3 FL, 1 MCL, and 1 MZL), AT101 showed a promising safety profile with 8.3% grade 3 CRS (*n* = 1) and 8.3% grade 4 ICANS (*n* = 1). In the whole cohort, the overall response rate was 91.7%, with a complete response rate of 75.0%, which improved to 100% in DL-2 and -3. AT101 expansion correlates with CR and B-cell aplasia.

**Conclusions:**

We developed a novel, safe, and potent CART19 product that recognizes a membrane-proximal domain of CD19 with fast on- and off-rates and showed significant efficacy and promising safety in patients with relapsed B-cell NHL.

**Trial registration:**

NCT05338931; Date: 2022–04-01.

**Supplementary Information:**

The online version contains supplementary material available at 10.1186/s12943-023-01886-9.

## Background

CD19-directed chimeric antigen receptor T (CART19) cell therapies have shown impressive clinical outcomes in CD19^+^ B-cell malignancies. However, a significant fraction of patients still fails to a achieve complete response (CR) or eventually relapse after CART19, with different relapse rates based on specific diseases [1]. The quality of the interaction between CAR and CD19 on tumor cells is a critical factor governing CAR T cell function. Currently, all US FDA-approved commercial CART19 products, including tisagenlecleucel, lisocabtagene maraleucel, axicabtagene ciloleucel, and brexucabtagene autoleucel, use single-chain variable fragments (scFv) derived from the murine anti-human CD19 antibody FMC63 [2–4]. FMC63 recognizes an epitope in CD19 that is located between exons 2 and 4 [5]. Interestingly, several mechanisms of relapse include the loss of the FMC63-recognized CD19 epitope either through point mutations or epitope masking [6–8]; furthermore, ineffective CAR:CD19 interaction can lead to CART dysfunction [9–11].

CD19 FMC63-epitope loss can occur when CD19 is expressed on the surface of relapsed tumor cells, but the CD19 epitope is not available for CART19 recognition due to multiple causes. CD19 FMC63-epitope loss includes either mutation of the FMC63 epitope [12] or masking of that epitope by CAR19 itself [13]. Several CD19 point mutations have been reported in patients with diffuse large B cell lymphoma (DLBCL) and B-acute lymphoblastic leukemia (B-ALL) who showed resistance to CART19 cell therapy. Some of these mutations affect the FMC63-recognized epitope, leading to failure of FMC63-CART19 [8, 12, 14]. In addition, we previously identified a novel mechanism of resistance in which the CAR19 gene was inadvertently introduced into leukemic B-cell blasts during manufacturing, leading to the *in-cis* interaction of CAR19:CD19 leading to epitope masking and subsequent resistance [13]. Therefore, targeting epitopes that differ from FMC63 may potentially overcome FMC63-CART19-driven epitope loss.

Additionally, T cell dysfunction further contributes to FMC63-CART19 failure. While several factors contribute to this phenomenon, such as the baseline T cell quality (differentiation/ exhaustion) and the immunosuppressive microenvironment, the quality of the CAR:CD19 interaction also plays a critical role in determining the efficacy of CAR T immunotherapy [15]. For instance, it was shown that targeting a CD22 epitope that is closer to the cell membrane leads to improved immune synapse formation and superior in vivo CAR T anti-tumor effect as compared to targeting a membrane-distal epitope [16, 17]. Furthermore, we and others have demonstrated that modifying the structure of the CAR, for example, by shortening the linker between the variable regions of the scFv, can improve binding to the target by shortening the distance between CAR T cells and tumor cells, thus enhancing CAR T cell effector function [18]. Moreover, the on- and off-rates of the CAR:target interactions can determine the overall function of CAR T cells. It has been postulated that CARs with faster off-rates from tumor cells are more effective in providing prolonged CAR T cell function [11, 19].

To address the issues of CART19 relapse following FMC63-CART19 treatment, we hypothesized that: 1. a CART19 product targeting a non-FMC63 epitope of CD19 located closer to the cell membrane would overcome CD19-epitope loss and enhance T cell function; and 2. that a CART19 product characterized by a fast on- and off-rate would reduce T-cell AICD and dysfunction. To this goal, we developed a novel humanized antibody (h1218), characterized by the ability to bind to a membrane proxymal epitope of CD19 localized between amino acids 51–63 (exon 2), and characterized by fast on- and off-rates. Using the h1218 antibody, we generated a CAR19 containing the 4-1BB and CD3ζ intracellular signaling domains. We then functionally compared h1218-CART19 against the conventional FMC63-CART19 in preclinical models and upon promising preclinical activity, we moved the novel h1218-CART19 (AT101) to a multi-center phase I first-in-human clinical trial for patients with relapsed or refractory non-Hodgkin lymphomas. This clinical trial demonstrated promising complete response rates and manageable toxicities.

## Methods

### Anti-CD19 scFv screening and lead optimization

To develop a novel antibody against the extracellular domain (ECD) of human CD19 and different from the FMC63 antibody we performed competitive biopanning against a complex of recombinant human CD19 ECD (M1-P278) and the FMC63 scFv to block the FMC63 epitope, using a proprietary chicken immune library (Patent US16/768412). This library was generated by immunizing a chicken with human CD19. Potential binders were screened using periplasmic extracts and reconfirmed using cell-binding ELISA against CD19-positive (Raji) and -negative (K562) cell lines. The selected binders were cloned as recombinant scFv with a (G4S)3 linker, produced in HEK293F cells, and purified using a human Fc tag at the C-terminus of the constructs. The 1218 antibody was selected as the top CD19 binder based on the specific recognition of a non-FMC63 epitope. To optimize the affinity of 1218 scFv, six complementarity-determining regions (CDR) residues in the heavy and light chains were randomly mutated using NNK degenerate codons with 70% or more of the parent sequence. Binders with pre-specified affinities were selected based on CD19-positive (Raji) cell binding affinity (EC50 value). The optimized 1218 included three amino acid mutations in CDR3 of the parental light chain and showed a > 10-fold increase in EC50 compared to that of the wild-type clone. To minimize immunogenicity in humans, a humanized version of chicken 1218 scFv was obtained using CDR grafting. The human germline framework region donor V and I genes were selected using IMGT/V-QUEST; IGHV3-21*04 and IGHJ5*01 were employed as the V and J genes of the heavy chain, respectively; and IGLV1-51*02 and IGLJ2*01 were employed as the V and J genes of the light chain, respectively. The final h1218 scFv, including the humanized VL, (G4S)3 linker, and humanized VH, was synthesized using GenScript (Jiangsu, China) and translated into a protein using HEK293F cells.

### Epitope binning assay

The epitope binning assay of the h1218 antibody was performed using an 8-channel Octet system (Octet QKe, Pall ForteBio). AR2G (Amine Reactive 2^nd^ Generation, Pall ForteBio; Cat# 18–5092) biosensors were immobilized with 10 μg/mL FMC63 Fc and conjugated with 10 μg/mL CD19 ECD kappa light chain fusion protein (CD19-ECD-Ck) for 10 min. After stabilization, 10 μg/mL FMC63 or h1218 antibody was introduced and co-incubated with the FMC63-bound sensor chip for 10 min to evaluate additional binding. Optical Density (OD)_450_ nm was measured using an ELISA reader (Victor X3, PerkinElmer).

### Generation of CD19-mutated cell lines and epitope mapping mutagenesis assay

Human CD19-T2A-Green Fluorescent Protein (GFP) and cynomolgus CD19-T2A-GFP (Bioneer, Daejeon, Korea) mutants were subcloned into the pLenti6.3 plasmid (Invitrogen; Cat# K5315-20). Chimeric CD19 proteins constructed by overlap extension PCR were subcloned into the pLenti6.3 plasmid using human CD19-T2A-GFP and cynomolgus CD19-T2A-GFP plasmids. Overlap extension PCR method was also used to obtain pLenti6.3 plasmids for several CD19 mutants with point mutations in key codons. Lentiviral particles containing wild type (WT), chimeric, or mutant CD19 proteins were generated in LentiX 293 T cells. LentiX 293 T cells were transduced with a lentiviral vector, and transduced target cell lines were selected for blasticidin (Invitrogen; Cat# R210-01) before use as targets. Antibody binding to HEK293T cells expressing individual CD19 mutant variants was assessed by flow cytometry as measuring mean fluorescence intensity (MFI).

### Affinity measurement assay

The binding affinities of h1218 and FMC63 scFv were measured using the Octet system. Briefly, purified scFvs were immobilized on an AR2G chip (Amine Reactive 2^nd^ Generation, Pall ForteBio; Cat# 18–5092) surface using standard amine coupling. Recombinant human CD19-ECD-Ck (M1-P278) was injected over the chip at a constant flow rate at concentrations ranging from 12.5–400 nM for h1218 scFv and 50–1600 nM for FMC63. The association (on-rate) and dissociation (off-rate) rates of the protein complexes were monitored using OD_450_, and the data were fitted using a 1:1 model.

### Off-target scFv binding analysis: retrogenix assay

For CAR binding screening, h1218 and FMC63 scFv were fluorescently labeled and screened for binding to fixed HEK293 cells/slides transduced with 5,484 expression vectors encoding both ZsGreen1 and a full-length human plasma membrane protein or a cell-surface-tethered human secreted protein in duplicates (16 slide sets, *n* = 2 slides per slide set). In total, 16 primary hits (duplicate spots) were identified by analyzing fluorescence (Alexa Fluor 647 and ZsGreen1) using ImageQuant. There was a variety of intensities (signal to background), ranging from very weak to strong. A confirmation screen was performed by applying the h1218 scFv protein to all 16 hits, in duplicate, on new slides. Library and confirmation screenings, as well as analyses, were performed at Retrogenix, a part of the Charles River Laboratories.

### Generation of the CAR construct and preclinical lentiviral vector production

The standard FMC63-CAR (CTL019) includes FMC63 scFv, 4-1BB, and CD3ζ domains [20]. The h1218-CAR included h1218 scFv, 4-1BB, and CD3ζ domains. Replication-defective, third-generation lentiviral backbones (pTRPE or pLTG) were used for both constructs. Briefly, 8 × 10^6^ HEK293T cells were seeded per T175 culture flask in standard Roswell Park Memorial Institute medium 1640 (RPMI; Gibco; Cat# 11875–085) with 10% fetal bovine serum (FBS, Gibco; Cat# 16140–071) and incubated overnight at 37 °C. After 24 h, the HEK293T cells (~ 70–80% confluency) were transfected using a mixture of Lipofectamine 2000 (116 μL, Invitrogen), packaging plasmids (up to 18 μg of each), and 15 μg CAR plasmid per flask. Lipofectamine and plasmid DNA were diluted in 4.6 mL Opti-MEM Reduced Serum media (ThermoFisher) prior to transfer into lentiviral production flasks. At 24 h and 48 h following transfection, the culture media from each flask were collected, filtered (0.45 μm), and concentrated using high-speed ultracentrifugation (8,000 × g for 16–18 h or 25,000 × g for 2.5 h). Titration of the lentiviral titer was performed using HEK293T cells, and flow cytometry was performed using CAR-specific antibodies.

### Preclinical CAR T cell production

CAR T cells were generated as described previously [21]. Healthy primary human T cells were obtained from the Human Immunology Core of the University of Pennsylvania. CD4 and CD8 T cells were combined at a 1:1 ratio and activated in vitro using CD3/CD28 stimulatory Dynabeads (Gibco; Cat# 40203D) at a ratio of three beads/cell for 6 days. Lentiviral particles were added on the second day of activation at a multiplicity of infection (MOI) of 1.5. Cell counts and volumes were quantified every other day beginning on day 6 (after Dynabeads removal) using a Multisizer 3 Coulter Counter (Beckman). CAR T cells were frozen in 90% FBS/10% DMSO when the growth kinetics and cell size plateaued and the cell volume was ~ 350 fL. Prior to all functional assays, T cells were thawed and rested overnight at 37 °C in RPMI medium supplemented with 10% FBS.

### Cell lines and general cell culture

B-cell malignant cell lines (Raji, Nalm6, OCI-Ly18, Toledo, and Pfeiffer) were originally purchased from the American Type Culture Collection (ATCC) or the Deutsche Sammlung von Mikroorganismen und Zellkulturen (DSMZ). The LentiX 293 T cell line was obtained from Takara Bio. All cell lines were authenticated by short tandem repeat (STR) analysis (University of Arizona) and tested for mycoplasma contamination every two months (Lonza; Cat# LT07-710). The cell lines were maintained in R10 medium (RPMI medium 1640 supplemented with 10% FBS, 1% GlutaMAX supplement (Gibco; Cat# 35050–079), 1% Pen/Strep (Gibco; 15140–122) and 1% HEPES (Gibco; Cat# 15630–130)) at 37 °C in 5% ambient CO_2_.

### Flow cytometry

Cells isolated from in vitro or in vivo assays were stained with antibodies for 15 min in the dark and washed once with phosphate-buffered saline (PBS; Gibco; Cat# 10010031) supplemented with 2% FBS. To determine the absolute cell numbers (e.g., tumor or T cells) acquired during flow cytometry, Flow-Count Fluorospheres (Beckman Coulter; Cat# 754053) were used according to the manufacturer’s protocol. To monitor caspase 3/7 activity, CellEvent Caspse3/7 Green Read Flow (Invitrogen; Cat# C10427) was used according to the manufacturer’s protocol. In all analyses, the population of interest was gated based on the forward versus side scatter characteristics, followed by singlet gating. Live cells were gated using ViaKrome 808 (Beckman Coulter; Cat# C36628). The surface expression of FMC63-CAR19 was detected using an anti-idiotype PE-conjugated antibody (Novartis). Flow cytometry was performed using a 6-laser Cytoflex LS Flow Cytometer (Beckman Coulter). All data analyses were performed using FlowJo 9.0, or 10 software (FlowJo, LLC, BD).

### Cytotoxicity assays

The target cells were transduced with a Click Beetle Green luciferase (CBGLuc)-GFP lentiviral construct and sorted using a FACS Melody Cell Sorter (BD). For short-term killing assays, target cells and CAR T cells or controls were incubated according to the manufacturer’s protocol at indicated E:T ratios. D-luciferin potassium salt (Gold Biotechnology; Cat# 115144–35-9) was diluted in PBS to a final concentration of 15 mg/mL and incubated together with the cell culture at 37 °C for 10 min. Cell survival was quantified as bioluminescent intensity using a BioTek Synergy H4 imager, and signals were analyzed using the BioTek Gen5 software (Agilent Technologies). Percent specific lysis was calculated using the control of target cells without effectors.

For long-term killing assays, CAR T cells were combined with target cancer cells at an E:T ratio of 0.125:1 or 0.0625:1, and co-cultures were evaluated for absolute counts of T cells and cancer cells by flow cytometry using CountBright absolute counting beads (ThermoFisher; Cat# C36950) every 48–72 h. The cultures were maintained at a concentration of 1 × 10^6^ cells/mL.

### Cytokine secretion assay

Target and effector cells were incubated at a ratio of 5:1 or 3:1 in R10 medium for 24 h. Supernatants were collected and stored at -80 °C if not used immediately. Analyses were performed using ELISA MAX standard set human IL-2 (BioLegend; Cat# 431801) or human IFN-γ (BioLegend; Cat# 430101 and BD Biosciences; Cat# 555142) kits.

### Cell avidity measurement using z-Movi

CAR T cell binding avidity against the Nalm6 monolayer was measured using a z-Movi Cell Avidity Analyzer (LUMICKS). Briefly, z-Movi-compatible acoustofluidic chips were coated with poly-L-lysine (Sigma-Aldrich; Cat# P4707) for 3 h prior to attaching a monolayer of tumor cells. 2 × 10^6^ cells Nalm6 cells were seeded on each z-Movi chip at a concentration of 1 × 10^8^ cells/mL and cultured at 37 °C in a dry incubator, 2 h in advance. CAR T cells (1 × 10^5^ CAR T cells stained with CellTracker Deep Red Dye (ThermoFisher; Cat# C34565) were introduced and incubated on the Nalm6 monolayer for 15 min. An acoustic force was applied, and cell detachment was measured using ImageJ and R. Avidity experiments were conducted according to the manufacturer (LUMICKS) instructions and recommendations, and cell avidity was quantified using the z-Movi software (v1.0).

### Xenograft mouse models

Six- to 8-week-old NOD-SCID gamma chain deficient (NSG, NOD.Cg-Prkdcscid Il2rgtm1Wjl/SzJ) mice were obtained from the *Stem Cell and Xenograft Core* at the University of Pennsylvania. The NSG mice were housed under pathogen-free conditions. The cancer cell lines (Raji and Nalm6) were engineered to express CBGLuc. 1 × 10^6^ Nalm6 cells were injected through the tail vein in 0.15 mL sterile PBS. Tumor growth was monitored over time using a Xenogen IVIS bioluminescence imaging system (PerkinElmer) and quantified by bioluminescence intensity (BLI) 10 min after luciferin injection (100 mg/kg) (GoldBio; Cat#: LUCK-1G). Five to seven days after tumor cell injection, when the bioluminescence intensity (BLI) reached ~ 10^7^ (total flux [P/S]), the selected amount of CAR ^+^ T cells or control untransduced T cells (UTD) were injected via the tail vein into tumor-bearing mice. The animals were monitored for signs of disease progression and overt toxicity, such as xenogeneic graft-versus-host disease, as evidenced by a > 10% loss in body weight, fur loss, diarrhea, conjunctivitis, and disease-related hind limb paralysis. NIH Guidelines were followed for animal care and use. All experimental protocols were approved by the University of Pennsylvania Institutional Animal Care and Use Committee (IACUC).

### Confocal microscopy imaging of the immune synapse

Planar lipid bilayers were prepared by fusing small liposome droplets on clean glass coverslips as previously described [22]. Briefly, the liposome was trapped in a μ-Slide VI 0.4 chamber (Ibidi; Cat# 80606). Lipid bilayers were first blocked with 5% casein for 20 min and then incubated with 6.3 nM streptavidin (ThermoFisher; Cat# 434301) for 15 min. After washing with imaging buffer (HEPES-buffered saline), lipids were incubated with biotinylated CD19 protein (R&D Systems; Cat#AVI9269) conjugated with Alexa Fluor 647 at room temperature for 30 min. After washing with the imaging buffer, lipid bilayers were blocked with 2.5 μM D-biotin to saturate the streptavidin-binding sites. CAR T cells were then stimulated on the lipid bilayers for 2 h at 37 °C. The cells were fixed in freshly prepared 4% paraformaldehyde for 15 min and permeabilized in 0.5% Triton-X100 and 10% normal donkey serum in PBS for 30 min at room temperature. The cells were then stained with primary antibodies against perforin (dG9, BioLegend; Cat# 308105), pCD3ζ (phospho Y83, Abcam; Cat# ab68236), and pZAP70 (Tyr318, Cell Signaling; Cat# 2701), as described previously [23]. F-actin was stained with Alexa Fluor 405-conjugated phalloidin (ThermoFisher; Cat# A30104). A Nikon A1R HD confocal microscope was used to obtain confocal images.

### General preclinical work statistical analysis

All in vitro data are representative of at least two independent experiments. All results are represented as either individual values or the mean ± standard error of the mean (SEM). Comparisons between the two groups were performed using a two-tailed unpaired Student's t-test. Comparisons between more than two groups were performed using one-way analysis of variance (ANOVA) with Tukey’s correction for multiple comparisons unless otherwise noted. Survival data were analyzed using the log-rank (Mantel-Cox) test. The *p*-values are denoted by asterisks as follows: * *p* < 0.05, ** *p* < 0.01, *** *p* < 0.001, and **** *p* < 0.0001. Data analysis was performed using GraphPad Prism v9.0 (San Diego, CA).

### Clinical trial design

This multicenter, non-randomized, open-label, phase I study was conducted at two centers in South Korea. The study was design was 3 + 3 dose escalation. The inclusion and exclusion criteria are described in the main text. Following leukapheresis and CAR T cell manufacturing, patients received lymphodepletion (LD) with intravenous fludarabine (25 mg/m^2^, on days -4, -3, and -2) and cyclophosphamide (250 mg/m^2^, on days -4, -3, and -2). The LD regimen used was identical to the one approved by the FDA for CART19 product, tisagenlecleucel [24]. Premedication with acetaminophen and diphenhydramine was administered 30–60 min before CAR T cell infusion. CAR T cells were administered as a single dose according to three dose levels. Lymphodepletion and CART infusion were administered in an inpatient setting, given the first-in-human nature of the study. Dose levels included 0.2 × 10^6^ cells/kg in DL-1, 1.0 × 10^6^ cells/kg in DL-2, and 5.0 × 10^6^ cells/kg in DL-3. Response assessment was performed at months 1, 3, 6, and 12 or when needed. Long-term follow-up was planned every 12 months for five years.

The primary endpoints were the safety and feasibility of CAR T cell manufacture. The secondary endpoints included efficacy, CAR T persistence, incidence and duration of B-cell aplasia and hypogammaglobulinemia, progression-free survival (PFS), and overall survival (OS) at one and two years.

The study was approved by the Ministry of Food and Drug Safety (MFDS) of Korea and the Research and Development Department of each participating center. This study was registered on ClinicalTrials.gov (NCT05338931). This study was conducted by AbClon Inc.. Written informed consent was obtained from all patients before study entry in accordance with the Declaration of Helsinki. This report incorporates data from all participants who received the AT101 before December 19, 2022. The last follow-up was conducted on September 25, 2023.

### Clinical-grade manufacturing of AT101 CART19 cells

The AT101 clinical phase I trial was conducted under the regulations of the Ministry of Food and Drug Safety (MFDS, South Korean regulatory agency). Clinical-grade AT101 was manufactured using an automated and closed system CliniMACS Prodigy (Miltenyi). Apheretic products were loaded onto the CliniMACS Prodigy using the TS520 tubing set, and T cells were enriched using the CliniMACS CD4 and CD8 selection process. A total of 5 × 10^7^ CD3^+^ T cells were loaded into CliniMACS Prodigy and expanded with anti-CD3/CD28 co-stimulation (TransAct, Miltenyi) and IL-2 supplementation (MACS GMP Recombinant Human IL-2, Miltenyi). T cells were transduced with clinical-grade h1218 lentivirus (Lentigen) (multiplicity of infection of 1) on day 1 and expanded to a maximum of 4 × 10^9^ cells. According to the MFDS regulations, quality control and release tests such as the Replication-Competent Lentivirus (RCL) assay, adventitious virus test, and sterility test for CAR T therapy products are performed using culture methods.

### Toxicity and response assessment

Cytokine-release syndrome (CRS) and immune-cell associated neurotoxicity syndrome (ICANS) were graded according to the American Society for Transplantation and Cellular Therapy criteria [25]. Adverse events were graded according to the Common Terminology Criteria for Adverse Events (version 5.0). PET-CT and CT/MRI data from lymphoma patients treated with AT101 were evaluated according to the Lugano criteria [26].

### Statistical analysis for the clinical trial

For statistical analysis, continuous variables are summarized as medians with ranges, unless otherwise specified and categorical variables as percentages. Progression-free survival was calculated as the time between the date of AT101 infusion and the event date (progression, death, or last follow-up visit). Overall survival was calculated as the time between the date of AT101 infusion and the event date (death or last follow-up visit). Analyses and figures were performed using GraphPad Prism software 9.5.0 (730). The phase I clinical trial aimed to determine the maximum tolerated dose (MTD) and recommended phase II dose (RP2D) based on the safety and tolerability of AT101; therefore, a statistical hypothesis test was not conducted.

## Results

### Identification of h1218, a novel humanized chicken scFv recognizing a membrane-proximal epitope of human CD19

**Fig. 1:**
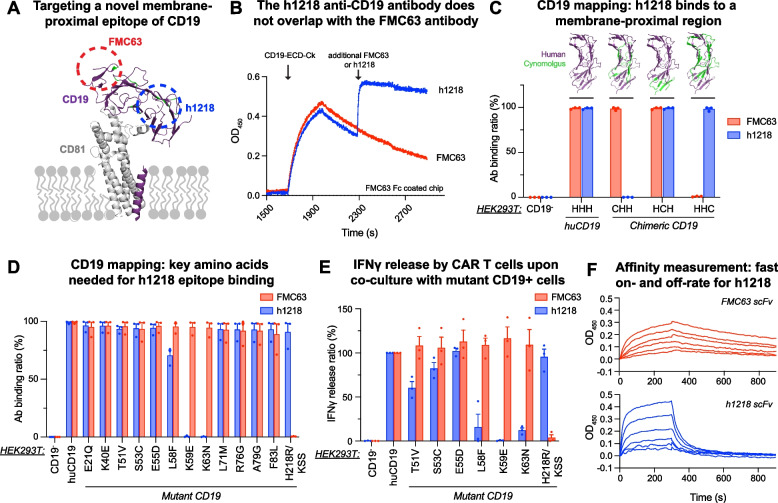
The h1218 antibody is specific for CD19 and recognizes a non-FMC63 membrane proximal epitope. **A** Schematic of FMC63 and h1218 antibodies binding sites on CD19. **B** Binding of h1218 to FMC63 bound-human CD19 complex. Sensor chips were coated with FMC63 Fc and CD19-ECD-Ck from 1700 to 2300 s. Additional FMC63 or h1218 antibody was added to the FMC63-bound sensor chip at 2300 s and monitored for further binding activities. **C** h1218 antibody binding test on wild-type HEK293T (CD19 negative), HEK293T cell expressing human CD19 (huCD19, HHH), and HEK293T cells expressing one of the three chimeric CD19 forms that had cynomolgus residues replacement at different region of CD19, respectively (CHH, HCH, and HHC). **D** Mutagenesis study to identify key residues corresponding to the h1218 CD19 epitope. **E** Quantification of IFNγ release on HEK293T cells expressing WT or mutant CD19 to identify additional key residues corresponding to the h1218 CD19 epitope. **F** Binding affinity of FMC63 and h1218 scFv to recombinant human CD19-ECD-Ck (M1-P278). Each line represents affinity measured at a different scFv concentration. All the experiments were repeated at least twice

We first sought to identify an antibody against a CD19 epitope that was distinct from the one recognized by the FMC63 antibody that is used in all the currently FDA-approved CART19 (Fig. 1A). To this goal, we screened a proprietary chicken immune single-chain variable fragment (scFv) library against the human CD19 extracellular domain (M1-K291). Given the greater evolutionary distance between humans and chickens as compared to humans and mice, the use of a chicken library allows for the generation of antibodies recognizing unique epitopes compared to murine-developed antibodies. The initial screening included six hits, from which we selected clone 1218, which was characterized by the absence of competition with FMC63 for CD19 (M1-P278). Indeed, in epitope binning experiments using the Octet assay, we showed that FMC63 and h1218 could simultaneously bind to CD19 (Fig. 1B), confirming that they recognize distinct epitopes. The chicken 1218 scFv was humanized by CDR-grafting to human germline genes and backmutations, generating the humanized 1218 scFv (h1218) (Figure S1A). To define the epitope recognized by the 1218 scFv, we performed domain- and epitope-mapping mutagenesis. We divided the extracellular portion of CD19 into three domains and replaced each human (H) domain with cynomolgus (C) monkey domains obtaining the following chimeras: H–H-H-(cell), C-H–H-(cell), H-C-H-(cell), and H–H-C-(cell) (Table S1). The h1218 scFv was able to recognize H–H-C, but not C-H–H, suggesting that, unlike FMC63 scFv, h1218 scFv recognizes an epitope in the most membrane-proximal domain between amino acids 51 and 63 (Fig. 1C). To further characterize the specific epitope, we mutated single amino acid codons and found that the key amino acids required for the binding of h1218 scFv were L58, K59, and K63. Conversely, the membrane-distal amino acid H218/KSS [27] was recognized as a key residue for FMC63 binding (Fig. 1D). ELISA further confirmed this characterization by revealing a significant reduction in interferon-gamma production in h1218-CART19 cells upon mutation of the amino acids L58, K59, and K63. Mutations in amino acids T51, S53, and E55 also influenced this production, albeit to a lesser extent (Fig. 1E). Importantly, we established that the h1218 scFv had a similar affinity to CD19-ECD-Ck as compared to FMC63 scFv (mean K_D_, 1.85 × 10^−7^ M and 1.49 × 10^−7^ M, respectively), but the binding kinetics of the two antibodies differed significantly (Fig. 1F). Indeed, the h1218 scFv had faster binding to the target (on-rate) ability (mean K_on_, FMC63 = 8.94 × 10^3^(1/Ms) versus h1218 = 8.68 × 10^4^(1/Ms)) and a significantly faster off-rate (mean K_off_, FMC63 = 1.31 × 10^–3^(1/s) versus h1218 = 1.60 × 10^–4^(1/s)).

Lastly, to confirm the specificity of the h1218 scFv to CD19, we screened for binding interactions against HEK293 cells expressing 5,484 human plasma membrane proteins or cell surface-tethered human-secreted proteins (*Retrogenix assay*). We observed high-intensity binding to CD19, together with non-specific lower-intensity binding to three additional proteins (TMEM108, SUSD5, and GUCA2A) (Figure S1B). To test whether the h1218 scFv was able to functionally recognize only CD19 and not these three possible off-targets, we performed flow cytometry and ELISA to test the binding of h1218 to HEK293T cells overexpressing TMEM108, SUSD5, or GUCA2A. The results showed no activation of h1218-CART19 by these targets (Figure S1C-E). Therefore, we excluded any non-specific binding to TMEM108, SUSD5, or GUCU2A.

### h1218-CART19 cells overcome CD19-FMC63 epitope loss resistance in preclinical models

**Fig. 2:**
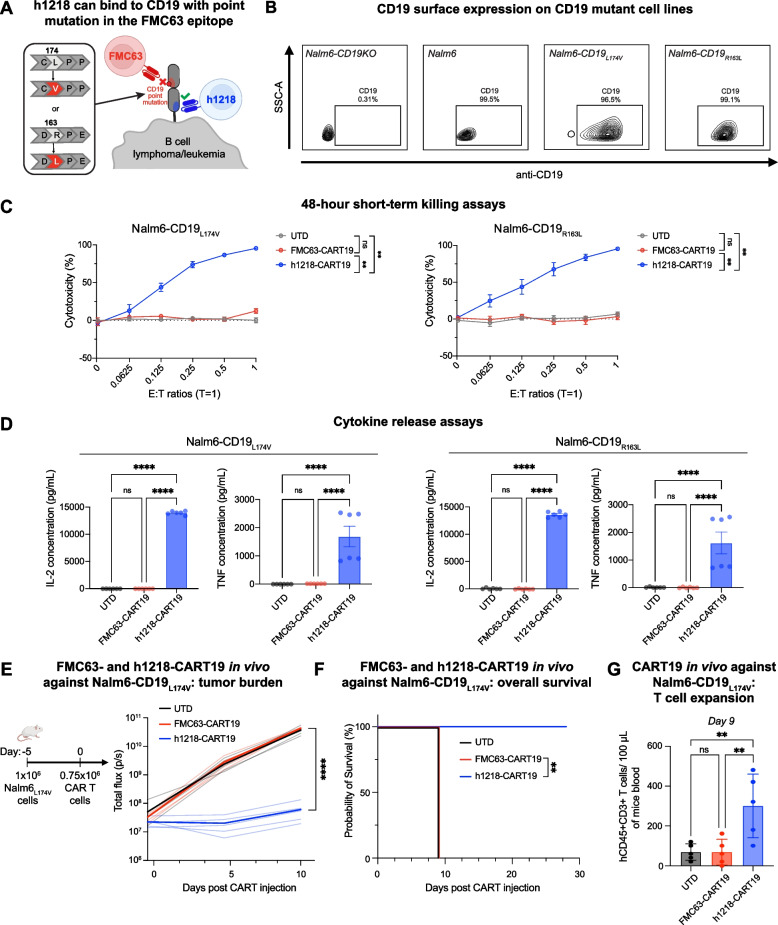
h1218-CART19 recognize and kill malignant B cells carrying FMC63-resistant CD19 mutations. **A** Schematic representation of B cell leukemia cells with point mutations in the membrane-distal CD19 domain (CD19_R163L_ or CD19_L174V_) as clinically identified post FMC63-CART19 treatment. **B** CD19 expression levels in Nalm6-CD19 KO, Nalm6 (wild type), Nalm6-CD19_L174V_ (left) and Nalm6-CD19_R163L_ (right) cell lines as measured by flow cytometry. **C** CART19 cytotoxicity against Nalm6-CD19_L174V_ and Nalm6-CD19_R163L_ at various effector to target (E:T) ratios (*n* = 3 independent donors) (luciferase assay). **D** IL-2 and TNF cytokine release measured by ELISA 24 h after CART19 and cancer cell co-culture at an E:T ratio of 5:1 (*n* = 2 donors). All values determined to be negative by comparison with the standard curve are shown as zero. **E** (Left) Schematic of the xenograft NSG mouse model: 1 × 10^6^ luciferase ^+^ Nalm6-CD19_L174V_ cells and 0.75 × 10^6^ UTD, FMC63-CART19, or h1218-CART19 cells were intravenous injected with a 5-day interval. (Right) Tumor burden of engrafted mice treated with UTD (*n* = 5), FMC63-CART19 (*n* = 5), or h1218-CART19 (*n* = 5) as measured by bioluminescence imaging. The bold lines represent the median luminescence of each group. **F** Overall survival in each treatment group (*p* = 0.0027). **G** Absolute cell counts of huCD45^+^ huCD3^+^ T cells in 100μL mouse blood on day 9. All bar graphs and cytotoxicity curves are presented as the mean ± SEM. Survival curves were compared using the log-rank (Mantel-Cox) test, and one-way ANOVA was performed with Tukey’s correction for multiple comparisons; **** *p* < 0.0001, *** *p* < 0.001, ** *p* < 0.01, and * *p* < 0.05. All bar graphs are presented as the mean ± SEM. All experiments were repeated at least twice

Having developed a novel scFv targeting a membrane-proximal epitope of CD19, we sought to develop a second-generation CAR construct. We used lentiviral backbones (pTRPE or pLTG) and included a CD8α hinge and transmembrane domain, together with 4-1BB costimulatory and CD3ζ signaling domains. We used this construct to generate lentiviruses and transduce and expand human T cells as previously described [20, 28]. We first tested this new product (h1218-CART19) in FMC63-CART19-resistant models. Two point mutations in exon 3 of CD19 (R163L and L174V) have been described in post-FMC63-CART19 biopsies, which drive FMC63-CART19 resistance and lead to FMC63 CD19 epitope loss [8, 14]. To model CD19-mutant escape, we used CRISPR-Cas9 technology (Table S2) to knock out wild-type CD19 in the standard B-ALL cell line Nalm6 (Nalm6-CD19KO), and subsequently transduced these cells with lentiviral constructs harboring one of the two reported point mutations (Nalm6-CD19_R163L_ or Nalm6-CD19_L174V_) (Fig. 2A). The surface expression of the CD19 mutants was confirmed by flow cytometry (Fig. 2B). We then evaluated the cytotoxic capacity of control untransduced (UTD) T cells, FMC63-CART19, and h1218-CART19 in vitro against the two engineered cell lines and found that h1218-CART19, but not FMC63-CART19, could recognize and target both Nalm6-CD19_R163L_ and Nalm6-CD19_L174V_ cells in short-term killing assays (Fig. 2C). Consistent with these findings, h1218-CART19 cells secreted IL-2 and TNF in the presence of both cell lines, whereas FMC63-CART19 cells did not (*p* < 0.0001 for both cytokines) (Fig. 2D). To confirm these results in vivo, we tested the efficacy of FMC63- and h1218-CART19 against the more frequently reported Nalm6-CD19_L174V_. We engrafted  immunodeficient NOD-SCID gamma chain deficient (NSG) mice with 1 × 10^6^ Nalm6-CD19_L174V_ cells on day -5 and randomized the mice to receive 0.75 × 10^6^ UTD, FMC63-CART19, or h1218-CART19 T cells on day 0 (Fig. 2E). Notably, h1218-CART19-treated mice showed significant efficacy in terms of both tumor control and overall survival (median overall survival, FMC63-CART19 = 9 days versus h1218-CART19 = not reached, *p* = 0.0027) (Fig. 2E-F). Furthermore, the in vivo expansion of h1218-CART19 cells in the blood was also significantly higher than that in the controls (mean T cell count, FMC63 = 68 cells/100 µL blood versus h1218 = 300 cells/100 µL blood, *p* = 0.0096) (Fig. 2G). These results demonstrated the ability of h1218-CART19 to overcome FMC63-resistant CD19 mutations.

**Fig. 3:**
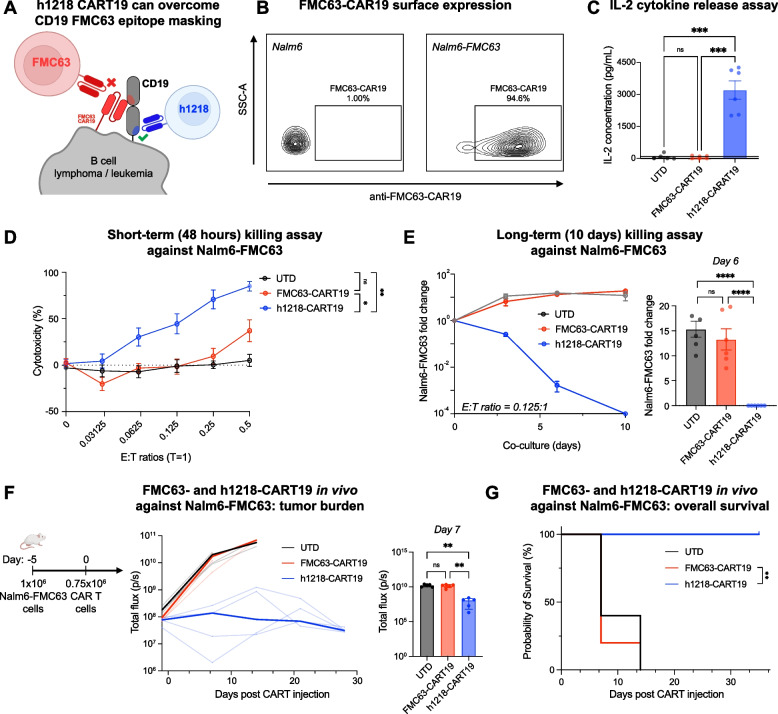
h1218-CART19 recognize and kill relapsed FMC63-CAR19^+^ Nalm6. **A** Schematic representation of a B cell lymphoma/leukemia cell accidentally transduced with the FMC63-CAR19 lentivirus during manufacturing that leads to epitope masking and resistance to FMC63-based CART19. **B** FMC63 expression level on Nalm6 cells measured by flow cytometry. **C** IL-2 cytokine release quantification by ELISA 24 h after CART and cancer cell co-culture at a E:T ratio of 5:1 (*n* = 2 donors). All values determined as negative by comparison to the standard curve are shown as zero. **D** 48-h CART cytotoxicity against Nalm6-FMC63 at various E:T ratios (*n* = 2 donors) by flow cytometry. **E** Tumor growth in the presence of CART cells over 10 days (*n* = 3 donors) by flow cytometry. (Left) Fold change over time of Nalm6-FMC63 cells compared to day 0 counts. (Right) Nalm6-FMC63 fold change on day 6. **F** (Left) Schematic of the xenograft mouse model: 1 × 10^6^ luciferase^+^ Nalm6-FMC63 cancer cells were engrafted 5 days before intravenous injection of 0.75 × 10^6^ UTD or CART cells. (Right) Tumor burden over time in mice bearing Nalm6-FMC63 with UTD (*n* = 5), FMC63-CART19 (*n* = 5), or h1218-CART19 (*n* = 5). Bolded line represents the median of each group. **G.** Overall survival in mice bearing Nalm6-FMC63 (*p* = 0.0016). All bar graphs and cytotoxicity curves are represented as mean ± SEM. One-way ANOVA was performed with Tukey correction for multiple comparisons; survival curves were compared using the log-rank (Mantel-Cox) test; **** *p* < 0.0001, *** *p* < 0.001, ** *p* < 0.01, and * *p* < 0.05. All bar graphs are represented as mean ± SEM. All the experiments were repeated at least twice

Another unique, although rare, mechanism of FMC63-CD19 epitope loss is the accidental transduction of the *FMC63-CAR19* gene into leukemic B-cell blasts, which causes CD19 epitope masking due to the *in cis* CAR19:CD19 interaction. We previously demonstrated that CAR19^+^ B-ALL blasts become resistant to FMC63-CART19 cells despite the fact that CD19 is still expressed on the surface [12]. We speculated that h1218-CART19 would be able to recognize these cells, given that they recognize a different CD19 epitope not "masked" by the FMC63-CAR19. Therefore, we used CD19^+^ Nalm6 cells transduced with a lentiviral vector carrying FMC63-CAR19 expressing both CD19 and FMC63-CAR19 (Fig. 3A-B). These cells were previously shown to be resistant to FMC63-CART19 [12]. In preclinical in vitro models, h1218-CART19 cells showed effective cytotoxicity in the short- and long-term and cytokine secretion (IL2) against Nalm6-FMC63 cells, whereas FMC63-CART19 cells demonstrated no activity (Fig. 3C-E). To confirm these results in vivo, we tested the efficacy of h1218-CART19 against FMC63-CD19-epitope-masked cancer cells by engrafting immunodeficient NSG mice with 1 × 10^6^ Nalm6-FMC63 cells on day -5 and randomized the mice to receive 0.75 × 10^6^ UTD, FMC63-CART19, or h1218-CART19 T cells on day 0 (Fig. 3F). Notably, all h1218-CART19-treated mice exhibited significantly enhanced tumor control and longer overall survival than FMC63-CART19-treated mice (median overall survival, FMC63-CART19 = 7 days versus h1218-CART19 = not reached, *p* = 0.0016) (Fig. 3F-G). These results suggest that the novel h1218-CART19 product, targeting a membrane-proximal epitope of CD19, can recognize and respond to cancer cells that have relapsed after CART19 with mutations in CD19 or epitope masking.

### h1218-CART19 cells demonstrate greater efficacy than FMC63-CART19 cells in human preclinical models of B-cell neoplasms.

**Fig. 4:**
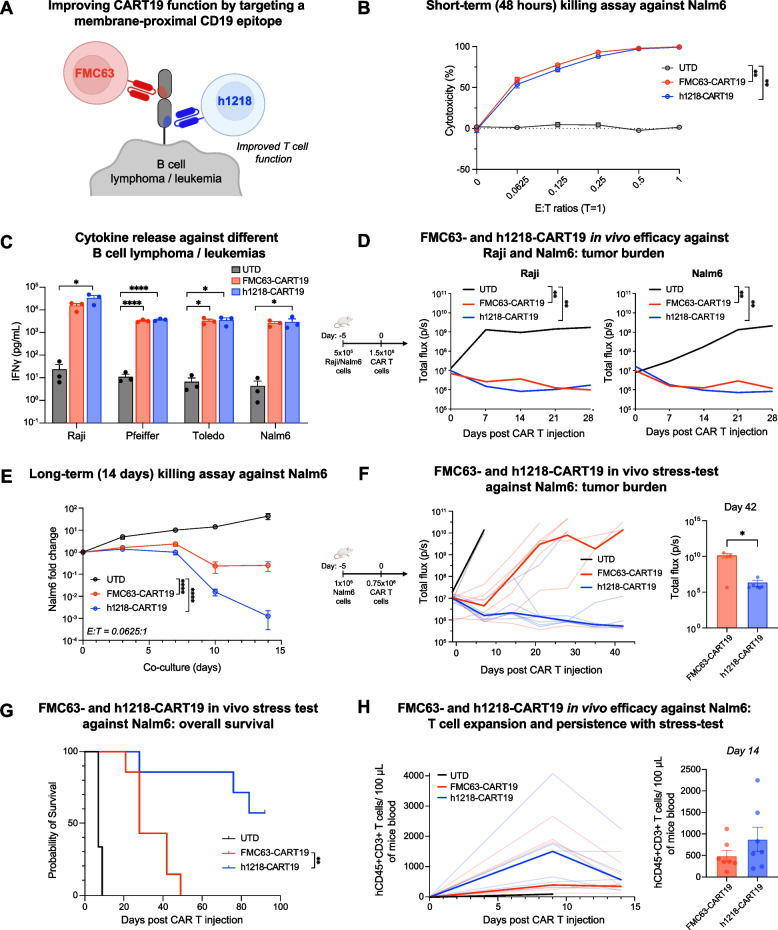
h1218-CART19 demonstrates enhanced efficacy than FMC63-CART19. **A** Schematic of h1218-CART19 targeting the membrane-proximal domain of CD19 in B cell lymphoma/leukemia as compared to standard FMC63-CART19. **B** Nalm6 tumor killing by UTD, h1218-CART19, or FMC63-CART19 cells at various E:T ratios (*n* = 3 donors) by flow cytometry. **C** IFNγ release as measured by ELISA upon stimulation of CD19-expressing tumor cells (Raji, Pfeiffer, Toledo, and Nalm6) at an E:T ratio of 3:1 (*n* = 2 donors). **D** (Left) Schematic of the in vivo experiment: either 1.5 × 10^6^ UTD or CART19 cells were infused 7 days after intravenous injection of  luciferase ^+^ Raji or lNalm6 cells. Tumor progression in mice bearing Raji (middle) or Nalm6 (right) cells is shown. **E** Quantification of Nalm6 fold change over 14 days in the presence of UTD, FMC63-CART19, or h1218-CART19 at low-E:T ratio model (*n* = 2 donors) using flow cytometry. **F** (Left) Schematic of the in vivo xenograft model: 0.75 × 10^6^ UTD or CAR T cells were infused intravenously 5 days after luciferase^+^ Nalm6 engraftment. (Middle) Tumor progression over time in mice bearing Nalm6 cells treated with UTD (*n* = 3), FMC63-CART19 (*n* = 7), or h1218-CART19 (*n* = 7) measured by luminescence. Bolded lines represent the median tumor burden in the corresponding group. (Right) Tumor burden on day 42 after CART19 injection. **G** Overall survival in mice bearing Nalm6. **H** (Left) CAR T cell expansion kinetics in the peripheral blood after CART19 injection. Bolded lines represent the median CAR T expansion. (Right) Quantification of CAR T cells in the blood 14 days after CART19 injection using flow cytometry. All bar graphs and cytotoxicity curves are presented as the mean ± SEM. Student's t-test was used to compare two groups; one-way ANOVA was performed with Tukey’s correction for multiple comparisons; survival curves were compared using the log-rank (Mantel-Cox) test; **** *p* < 0.0001, *** *p* < 0.001, ** *p* < 0.01, and * *p* < 0.05. All bar graphs are presented as the mean ± SEM. All experiments were repeated at least twice

As previously discussed, the h1218 scFv is characterized by faster on- and off-rates than those of the FMC63 scFv. We hypothesized that the h1218 CAR could lead to improved outcomes compared to standard FMC63-CART19 by reducing activation-induced cell death (AICD) upon antigen engagement (Fig. 4A). To test this hypothesis, we assessed h1218-CART19 and FMC63-CART19 activities in clinically relevant preclinical models of NHL/ALL in vitro and in vivo. In the short-term (48 h) in vitro killing assays, FMC63- and h1218-CART19 cells demonstrated similar cytotoxic effects against Nalm6 and other hematological malignancies (Fig. 4B; Figure S2A-B). This result was consistent with the fact that in the short-term, there was no significant difference in IFNγ cytokine release levels between FMC63- and h1218-CART19 cells against B-cell lymphoma/leukemia lines, including Raji (Burkitt lymphoma), Pfeiffer (DLBCL), Toledo (DLBCL), and Nalm6 (B-ALL) (Fig. 4C). In in vivo experiments, h1218-CART19 exhibited dose-dependent activity against Raji cells (Figure S3A-B), and when we used high doses of CAR T cells (1.5 × 10^6^), both FMC63-CART19 and h1218-CART19 had similar anti-tumor cytotoxicity to Raji and Nalm6 cells in vivo (Fig. 4D).

However, in "stress-test" experiments where we administered lower and sub-optimal CART19 doses (0.75 × 10^6^), we observed enhanced efficacy by h1218-CART19 cells compared to FMC63-CART19 both in vitro and in vivo. In particular, in a long-term in vitro killing assay with a low effector: target (E:T) ratio of 0.0625:1, h1218-CART19 showed improved tumor control against the B-ALL cell line Nalm6 compared to FMC63-CART19 (Fig. 4E). Furthermore, in an in vivo stress-test model of Nalm6 where only 0.75 × 10^6^ CAR^+^ T cells were injected into NSG mice, h1218-CART19 demonstrated significantly better tumor control than FMC63-CART19 (*p* = 0.042) (Fig. 4F; Figure S3C). This enhanced tumor control was also associated with longer overall survival (h1218-CART19 = not reached versus FMC63-CART19 = 28 days, *p* = 0.0017) (Fig. 4G). Lastly, we measured CART expansion and persistence in the blood of mice and observed higher expansion and persistence of h1218-CART19 than that of FMC63-CART19 (Fig. 4H). These results demonstrate that the novel h1218-CART19 has stronger preclinical anti-tumor activity than that of FMC63-CART19 and is associated with higher CART19 expansion and persistence.

### Faster on- and off-rates of h1218-CART19 cells are associated with decreased activation-induced cell death compared to FMC63-CART19

**Fig. 5:**
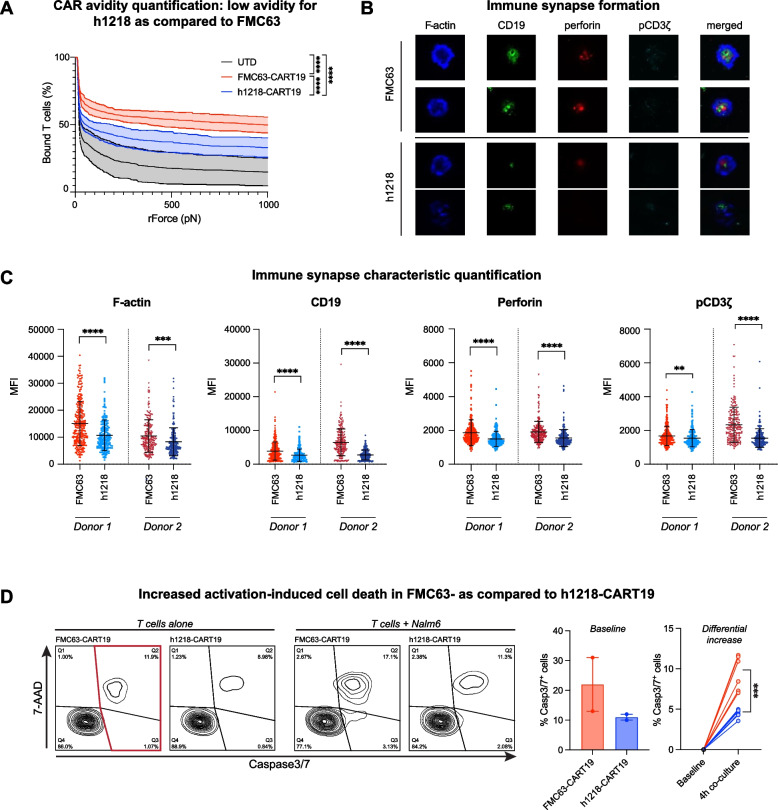
h1218-CART19 demonstrates lower avidity and less activation-induced cell death than FMC63-CART19. **A** Quantification of UTD, FMC63-CART19, and h1218-CART19 binding avidity to Nalm6 after 15-min co-culture (*n* = 2 donors) by Lumicks analysis. **B** Representative confocal microscopy images of F-actin, CD19, perforin, and phosphorylated-CD3ζ (pCD3ζ) (CAR) expressed in FMC63-CART19 or h1218-CART19 cells when engaged with biotinylated CD19 protein. **C** Quantification of F-actin, perforin polarization, and pCD3ζ in FMC63-CART19 or h1219-CART19 cells engaged with biotinylated CD19 protein (*n* = 2 donors). In total, 200 events were recorded for each group. **D** (Left) Representative flow cytometric analysis of Caspase3/7 ^+^ FMC63-CART19 and h1218-CART19 cells with and without 4-h stimulation by Nalm6. Caspase3/7 ^+^ population of CART19 cells is boxed in red. (Middle) Baseline level of Caspase3/7^ +^ population in FMC63- or h1218-CART19 cells at 0 h. (Right) Differential increase in the Caspase3/7^ +^ population after 4-h stimulation. All graphs are represented as the mean ± SEM. Student's t-test was used to compare two groups; one-way ANOVA was performed with Tukey’s correction for multiple comparisons; **** *p* < 0.0001, *** *p* < 0.001, ** *p* < 0.01, and * *p* < 0.05. All bar graphs are presented as the mean ± SEM. All experiments were repeated at least twice

To better understand the functional characteristics and mechanism that support the increased anti-tumor efficacy of h1218-CART19 versus FMC63-CART19, we next studied the cellular interaction between CART19 and tumor cells. Given the hypothesis that faster on- and off-rates of h1218 scFv are associated with enhanced CAR T function, we first quantified the cellular avidity of FMC63-CART19 and h1218-CART19 using the z-Movi platform (LUMICKS). In line with the previous affinity kinetics results of the scFv, h1218-CART19 cells showed reduced cellular avidity compared to FMC63-CART19 cells (mean avidity, FMC63 = 55.57% versus h1218 = 39.23%, *p* < 0.0001) (Fig. 5A). We then studied the immune synapse characteristics of different CAR T cells as previously described [29]. We quantified F-actin to measure the stability of the synapse and the clustering of CD19 to measure the physical synapse formation. In line with the lower avidity of h1218- as that of FMC63-CART19, we found that synaptic F-actin clustering and accumulation of CD19 protein were reduced in h1218-CART19 cells. We then studied early CAR signaling by measuring perforin and phosphorylated-CD3ζ (pCD3ζ) chain polarization to the synapse. Similarly, we observed less perforin and pCD3ζ at the immunological synapse at a 2-h stimulation time-point in h1218-CART19 cells than in FMC63-CART19 cells (Fig. 5B-C). These results are in line with the faster off-rate observed for h1218. Given the increased anti-tumor efficacy and in vivo proliferation of h1218-CART19 observed in our previous experiments, we hypothesized that h1218-CART19 was more efficacious than FMC63-CART19 owing to reduced early CAR T activation and AICD and subsequent higher survival of CAR T cells after tumor encounter. To prove this hypothesis, we measured AICD upon target encounters. Notably, h1218-CART19 showed lower caspase 3/7 cleavage at baseline and reduced AICD as measured by caspase 3/7 cleavage upon stimulation compared to FMC63-CART19 (Fig. 5D), suggesting higher levels of AICD in FMC63-CART19 cells, which may lead to decreased T cell numbers in the long-term.

### A first-in-human clinical trial of h1218-CART19 in relapsed and refractory NHL: study design and patients

Given the promising preclinical results of h1218-CART19, we sought to translate this preclinical product into a clinical good manufacturing product (GMP) product termed AT101 (AbClon Inc.). We started a phase I/II multi-center first-in-human clinical trial to establish the tolerability and the recommended phase II dose (RP2D) of AT101 in patients with B-NHL (NCT05338931). The study utilized a standard 3 + 3 dose escalation design (see “Methods” section) (Figure S4A). Here, we report the initial results of the Phase I portion of the trial. Key inclusion criteria comprised an established pathological diagnosis of B-cell non-Hodgkin lymphoma relapsed or refractory to at least one line of standard therapy; an ECOG performance status of 0–1, and adequate hematological, kidney, liver, lung, heart, and bone marrow functions. Patients previously treated with cellular therapies, autologous stem cell transplantation (ASCT), or CD3/CD20 bispecific antibodies were eligible for enrollment. Patients were initially screened for eligibility and signed an informed consent document before enrollment in the study. Autologous T cells were collected through standard apheresis and manufactured as AT101 using closed and automated systems. Patients were assigned a dose level upon the completion of apheresis. Upon receipt of the final AT101 product, the patients received lymphodepleting chemotherapy (LD) with intravenous fludarabine (25 mg/m^2^) and cyclophosphamide (250 mg/m^2^) on days -4, -3, and -2 prior to AT101 infusion. AT101 was administered as a single intravenous infusion at one of three dose levels (DL): DL-1:0.2 × 10^6^ cells/kg, DL-2:1.0 × 10^6^ cells/kg, and DL-3:5.0 × 10^6^ cells/kg. Patients were allowed to receive bridging therapy at the discretion of the principal investigator during AT101 manufacturing and up to 2 weeks before AT101 infusion.

As shown in the CONSORT diagram (Figure S4B), of 14 patients registered, 14 patients were enrolled, and 12 (85.7%) were infused. Two patients were not infused because of manufacturing failure due to insufficient CAR T expansion (*n* = 1; CAR T fold change on day 6 of expansion was 0.70) and bacterial contamination of the apheresis product due to asymptomatic bacteremia likely caused by bacterial translocation originating from an intestinal lesion (*n* = 1). AT101 CAR T products reached a median of 5.6 (5.2–6.0) population doublings at the end of manufacturing. The final median CD4/CD8 ratio was 0.8 (0.1–1.7), and 92% (86–95%) of the cells were CD45^ +^ CD3^ +^ T cells. The median frequency of CAR^ +^ cells among AT101 cells was 46% (25–57%) (Table S3). The median vein-to-vein time was 56 (48–97) days (Table S3), given that the current release and quality control (QC) tests (listed in the Methods) are performed using time-consuming culture methods.

Patient demographics for the 12 infused patients are summarized in Table 1 and detailed in Table S4. The median age was 62.5 (39–84) years, and 58.3% (7/12) of patients were female. The median number of previous lines of treatment was 3 (2–8). The non-Hodgkin lymphoma subtypes included diffuse large B-cell lymphoma (DLBCL, *n* = 7/12, 58.3%), follicular lymphoma (FL, *n* = 3/12, 25.0%), mantle cell lymphoma (MCL, *n* = 1/12, 8.3%), and marginal zone lymphoma (MZL, *n* = 1/12, 8.3%). Three patients had an ECOG score of 0, and nine patients of 1. Five patients (41.7%) had received a previous autologous stem cell transplantation (ASCT), one patient (8.3%) had received a non-gene edited NK cellular therapy, and three patients (25%) had received CD20/CD3 bispecific antibodies. Two patients (16.7%) had refractory disease. The median sum of the product of diameters (SPD) by CT scan was 1,041 (190–11,836) mm^2^. Two patients (6.7%, patient 9 and 12) received bridging therapy, in particular CHOP (cyclophosphamide, doxorubicin, vincristine, and prednisone) or ESHAP (etoposide, cisplatin, cytarabine, and methylprednisolone), between apheresis and CAR T infusion. Patient 9 did not respond to bridging therapy whereas patient 12 achieved a partial response. Five patients (41.7%) had elevated lactate dehydrogenase (LDH) levels. One patient had bulky disease (> 7 cm) (Table S4). Overall, this study enrolled patients who were heavily pretreated with, including novel T cell-based immunotherapies, but not CART19.

**Table 1:** Patient baseline characteristics

Baseline Characteristic	Value (%)
*Age, n (years)*	
< 60	5 (41.7)
≥ 60	7 (58.3)
Median (range)	62.5 (39–84)
*Histology, n (%)*	
DLBCL	7 (58.3)
FL	3 (25.0)
MCL	1 (8.3)
MZL	1 (8.3)
*Prior ASCT, n (%)*	5 (41.7)
*Number of prior lines of therapy, n (%)*	
≤ 2	5 (41.7)
≥ 3	7 (58.3)
Median (range)	3 (2–8)
*Refractoriness to prior chemotherapy, n (%)*	2 (16.7)
*Received bridging therapy*^*a*^*, n (%)*	2 (16.7)

*Abbreviations*:* DLBCL *diffuse large B cell lymphoma*, FL* follicular lymphoma*, MCL *mantle cell lymphoma, *MZL* marginal zone lymphoma, *ASCT *autologous stem cell transplantation

^a^Bridging was defined as any therapy received between leukapheresis and the start of LD chemotherapy

### Safety of h1218-CART19 in patients with relapsed or refractory B cell non-Hodgkin lymphoma

There were no infusion-related acute adverse events. Overall, 58.3% of the patients had at least one grade ≥ 3 adverse event (Table 2). As expected, neutropenia was the most common severe adverse event affecting seven patients and was likely related to LD chemotherapy. Among the 12 patients, six had a low B cell count before lymphodepletion (< 50 cells/μL). All patients experienced B cell aplasia following lymphodepletion and AT101 infusion. Patient 8, who had progressive disease (PD) at 3 months, recovered B cells at the same time as the disease progressed (Figure S5A). Grade 3 anemia was observed in four patients (33.3%), and grade ≥ 3 thrombocytopenia in two patients (16.7%). The levels of hemoglobin and platelets decreased early after lymphodepletion but promptly recovered in the responding patients (Figure S5B-C). Ferritin levels peaked during the first two weeks after AT101 infusion (Figure S5D). One patient (patient 12) experienced grade 3 sepsis on day 19 and promptly recovered with antimicrobial treatment; however, 27 days after the first infection, the patient developed new septic shock due to candidemia and died of multi-organ failure (Table 2, Table S4).

Cytokine-release syndrome (CRS) was observed in 33.3% of patients (*n* = 4), and it was severe (grade 3) in one patient (8.3%). No patients at DL-1 developed CRS, but three patients at DL-2/3 had grade 1 CRS, and one additional patient at DL-3 had grade 3 CRS. The median time of CRS onset was 6.5 (2–11) days after AT101 infusion and resolution happened within one day. None of the patients required steroids for the CRS management. Immune cell-related neurotoxicity syndrome (ICANS) occurred in 25% (*n* = 3) of patients, and it was severe (grade 4) in one patient (8.3%) at DL-1, which was the only dose-limiting toxicity (DLT) in this trial. The patient developed encephalopathy on day 12, which required intubation for airway protection. However, the patient recovered completely without neurological sequelae after six days of intravenous dexamethasone and intrathecal hydrocortisone treatment. Due to this DLT, 3 additional patients were enrolled in the DL-1 cohort without any further ICANS. One patient from the DL-2 cohort and one patient from the DL-3 cohort had grade 1–2 neurotoxicity, characterized by tremors and delirium, respectively. The median time of ICANS onset was 12 (8–12) days, with complete resolution within 3 (2–6) days with the use of dexamethasone (Table 2; Table S5).

**Table 2:** Summary of adverse events incidence, grade and duration by dose level

Adverse Event				
	***DL-1 (n = 6)***	***DL-2 (n = 3)***	***DL-3 (n = 3)***	***Total (n = 12)***
**Cytokine Release Syndrome (CRS)**				
**Any grade**	0	1	3	4 (33.3)
**Grade 1/2**	0	1	2	3 (25.0)
**Grade ≥ 3**	0	0	1	1 (8.3)
**Time to onset, days (range)**	-	11	2	6.5 (2–11)
**Time from onset to resolution, days (range)**	-	1	1	1 (1–1)
**Immune Cell Associated Neurotoxicity Syndrome (ICANS)**				
**Any grade**	1	1	1	3 (25.0)
**Grade 1/2**	0	1	1	2 (16.7)
**Grade ≥ 3**	1	0	0	1 (8.3)
**Time to onset, days (range)**	12	12	8	12 (8–12)
**Time from onset to resolution, days (range)**	6	2	3	3 (2–6)
**Other Adverse Events AE):**				
**Total Patients with Grade ≥ 3 AE**	4	1	2	7 (58.3)
**Neutropenia**	4	1	2	7 (58.3)
**Anemia**	3	0	1	4 (33.3)
**Thrombocytopenia**	1	0	1	2 (16.7)
**Infection**	1	0	1	2 (16.7)
v**Anorexia**	0	0	1	1 (8.3)
**Diarrhea**	0	0	1	1 (8.3)
**COVID-19**	0	1	0	1 (8.3)
**Hypokalemia**	0	0	1	1 (8.3)
**Pneumonia**	1	0	0	1 (8.3)
**Sepsis**	0	0	1	1 (8.3)

### Efficacy of h1218-CART19 in patients with relapsed or refractory B cell non-Hodgkin lymphoma

**Fig. 6:**
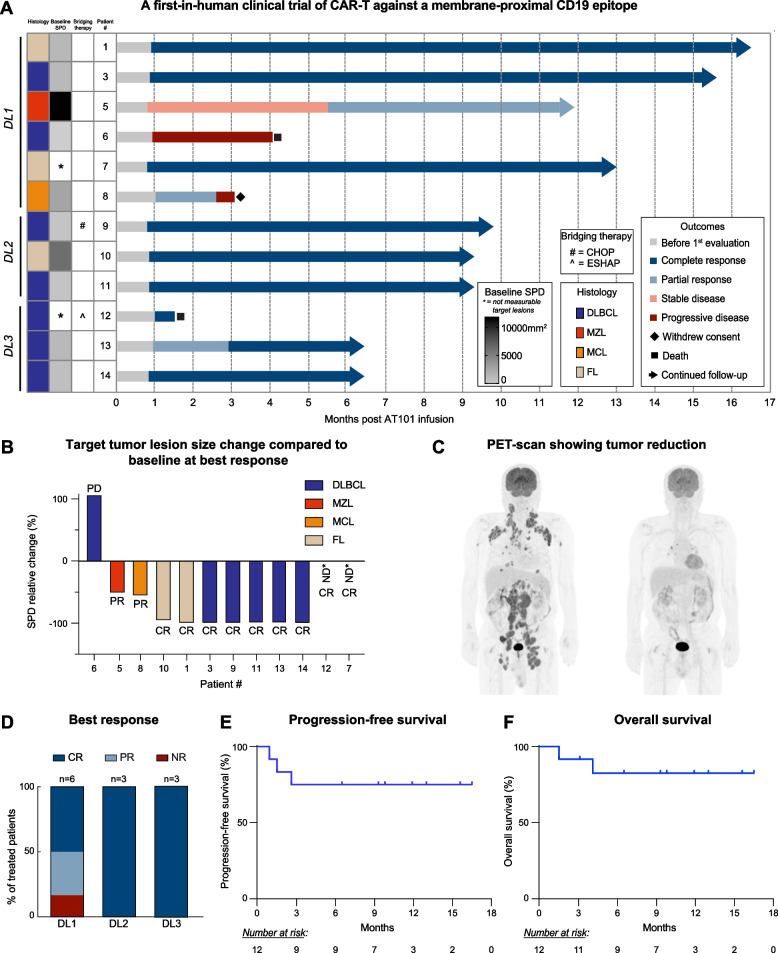
h1218-CART19 phase I clinical trial patient characteristics and response. **A** Swimmers' plot of individual patients with their responses to AT101 over time. Specific NHL subtype and baseline tumor burden is indicated (sum of the product of the diameters (SPD), mm^2^). See box legends for detail. Note: patients 7 had non-measurable disease involvement of sigmoid colon at time of AT101 infusion and patient 12 did not have a measurable target lesion after bridging therapy. **B** Waterfall plot depicting the change in tumor burden from baseline to the best response post-treatment for each patient. **C** Patient 10's PET/CT-scan before AT101 infusion and 1 month after AT101 infusion, showing complete metabolic response. **D** (Top) Best overall responses across all 12 patients. (Bottom) Best overall response by dose level (DL-1, DL-2, and DL-3). **E** Progression-free survival of patients treated with AT101. Patients at risk listed below. **F** Overall survival of patients treated with AT101. Patients at risk listed below

Of the 12 patients treated, one patient (8.3%) did not respond to AT101. All other patients responded with an overall response rate (ORR) of 91.7% (95%CI, 56.2–97.0), and a CR was observed in 10 patients (75%) (Fig. 6A-D). At month 1, the ORR was 83.3% (95%CI, 51.6–97.9), and CR was observed in 8 patients (66.7%). At month 3, of the 11 patients still alive, the 8 responding patients were all in CR (72.7%) (95%CI, 43.6–92.1) (Figure S6A). Of note, considering only the patients (*n* = 6) receiving higher doses of AT101 (DL-2 and DL-3), the CR was 100% (Fig. 6D). In the overall population, one patient (patient 8) with a partial response (PR) lost the response before month 3; all other responding patients (10/11) maintained their response, with one patient (patient 13) switching from PR to CR at month 3. With a median follow-up of 9.3 months (1.5–16.5 months), the progression-free survival (PFS) was 75.0%, and the overall survival (OS) was 82.5%. The median PFS and OS were not reached (Fig. 6E-F). One patient (patient 12) in CR died of septic shock. Median PFS and OS of patients achieving CR were not reached (Figure S6B-C). Based on these results, DL-3 was selected as the recommended phase-II dose.

### h1218-CART19 AT101 expansion, persistence, and serum cytokine levels

**Fig. 7:**
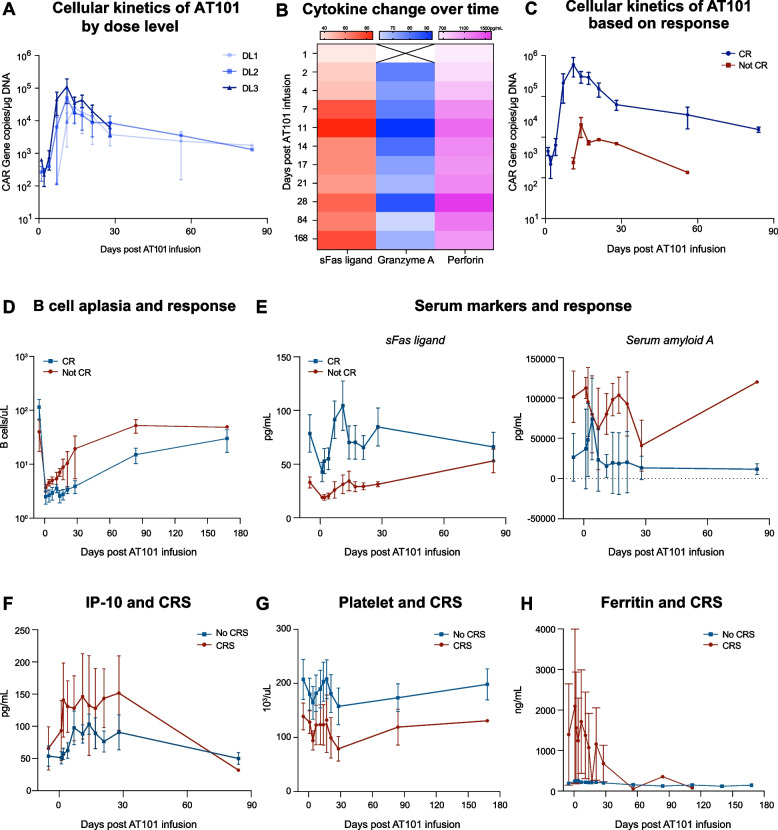
h1219-CART19 phase I clinical trial CAR T expansion, blood cell counts, and serum cytokine levels. **A** AT101 expansion and persistence in the blood detected by qPCR in DL-1, DL-2 and DL-3. **B** Mean serum levels of sFas ligand, granzyme A, or perforin across 12 patients over time by Luminex. **C** AT101 expansion in complete response patients (*n* = 9) and not complete response patients (*n* = 3) by qPCR. **D** B cell count in complete response patients (*n* = 9) and not complete response patients (*n* = 3). **E** (Left) sFas ligand and (right) serum amyloid A levels on day 1 pre AT101 infusion and days 2–168 post AT101 infusion in complete response patients and not-complete response patients by Luminex. **F** IP-10 serum level change over time in patients without CRS (*n* = 8) and patients with CRS (*n* = 4) by Luminex. **G** Platelet level change over time in patients without CRS (*n* = 8) and patients with CRS (*n* = 4) by Luminex. **H** Ferritin level change over time in patients without CRS (*n* = 8) and patients with CRS (*n* = 4)

To study the function of AT101 in patients and define possible correlates of response and toxicity, we measured CAR T kinetics in peripheral blood using flow cytometry and qPCR, and serum cytokine levels using the Luminex assay [30]. The AT101 transgene in the blood peaked on day 11 (11–14) after CAR T infusion. There was a correlation between peak expansion and infused dose (DL-1:2.5 ± 1.3, DL-2:5.0 ± 3.4, DL-3:113.6 ± 6.50 × 10^4^ CAR gene copies/µg DNA) (Fig. 7A). Furthermore, we measured cytokine levels in the serum at baseline and following AT101 infusion. Interestingly, in all patients, we observed an increase in key inflammatory cytokines after CAR T infusion, particularly sFas ligand, granzyme A, and perforin. The peaks of these cytokines were observed on day 11 (Fig. 7B; Figure S7).

We then investigated whether patients achieving CR displayed different proportions of CAR T transgenes and cytokine secretion in the peripheral blood. Indeed, the AT101 transgene peak was 6.0 × 10^4^ copies/μg DNA in patients who developed CR compared with 2.0 × 10^4^ copies/μg DNA in patients who did not develop CR (Fig. 7C). Similarly, reduced B cell levels after treatment and during follow-up were associated with CR (Fig. 7D). Additionally, sFas ligand levels were higher in CR patients than in non-CR patients. In contrast, serum amyloid A levels were higher in the non-CR group than in the CR group (Fig. 7E). Furthermore, patients with CRS had higher cytokine levels, particularly IP-10 (Fig. 7F), and lower platelet counts (Fig. 7G). Lastly, we observed higher peaks of ferritin in patients experiencing CRS as compared to patients with no CRS (Fig. 7H).

In summary, we developed a new CART19 product from bench to bedside and demonstrated that targeting a novel membrane-proximal epitope of CD19 with fast on- and off-rate CAR leads to a potent anti-tumor effect in patients with manageable toxicity.

## Discussion

CART19-directed immunotherapies have achieved remarkable efficacy in treating B-cell malignancies [13, 31, 32]. Unfortunately, the majority of patients treated with the currently available CART19 either do not respond or eventually relapse [33]. The goal of this study was to develop and clinically test a novel CART19 that targets a non-FMC63 epitope in CD19 with fast on- and off-rates to obtain higher efficacy and non-overlapping resistance mechanisms. Using a chicken library to target the human CD19 extracellular domain, we developed a novel anti-CD19 scFv (h1218) antibody that recognizes a membrane-proximal CD19 epitope characterized by low avidity. We demonstrated that this CART19 product, AT101, leads to an overall safe toxicity profile and a 100% response rate at the recommended phase II dose.

All four FDA-approved CART19 cells use the same scFv (derived from the FMC63 antibody) and recognize the same CD19 epitope. However, under certain circumstances, tumor cells can develop resistance mechanisms, leading to tumor escape and relapse [8, 12, 14, 34, 35]. In particular, loss or downregulation of the targeted antigen CD19 can negatively affect the functionality of CAR T cells both in vitro and in vivo as well as impair the long-term persistence of CAR T cells in vivo [36]. Previous studies have identified genetic mutations in CD19 exons 2–5 as the primary mechanisms responsible for CD19 loss [14]. In this study, we primarily focused on FMC63 epitope loss due to the development of point mutations in CD19, or masking of the epitope due to CAR19:CD19 *in cis* binding. Indeed, we demonstrated that h1218-CART19 possesses increased anti-tumor activity compared to conventional CART19 cells in preclinical leukemia and lymphoma models. We showed that h1218- but not FMC63-CART19 cells were able to recognize and kill FMC63-resistant cell lines that were generated to mimic real-world relapse settings.

In addition, T-cell dysfunction affects the ability of CAR T cells to survive and kill tumor cells in the immunosuppressive microenvironment [37]. We hypothesized that optimizing the interaction between the CAR and the target antigen would improve the ability of CAR T cells to survive and persist in vivo, leading to an enhanced anti-tumor function. Previous studies have suggested that lower affinity CARs in the presence of low antigen burden avoid excessive stimulation and promote less activation and exhaustion, leading to more durable anti-tumor responses in the long term [11, 38–40]. This effect was thought to be linked to the faster off-rates of the low-affinity CAR. In the context of h1218, we hypothesized that this might increase the ability of CART19 to perform “serial killing”, which is required for long-term tumor control [19]. Therefore, in addition to targeting a novel non-FMC63 CD19 epitope, a novel CAR with a similar affinity to FMC63-CAR, but with faster on- and off-rates, would lead to better anti-tumor effects. This would not only increase serial killing but also reduce the AICD of CAR T cells upon activation and ultimately enhance their overall function. Indeed, CAR T cells are susceptible to Fas- and DR5-dependent fratricidal AICD once the activation signaling threshold is surpassed, resulting in attenuated anti-tumor potency [41]. In previous studies, genetic engineering to incorporate a dominant negative Fas receptor in the CAR design, which disrupts the Fas-FasL interaction, has been shown to improve the anti-tumor activity and persistence of CAR T cells [42]. However, the elevation of inflammatory cytokine secretion and subsequent development of CART-related systemic toxicities have always been major concerns of this new approach [43]. Our study indicated that h1218-CART19 cells showed a reduced activation response when stimulated by CD19, and they demonstrated improved in vivo proliferation and tumor control ability in xenograft mouse models of B-cell acute leukemia compared with FMC63-CART19 cells. These results align with the understanding that faster off-rate CAR T cells have altered early activation levels in their transcriptomic and immunophenotypical profiles [39]. Most importantly, in this model, we did not observe a significant increase in cytokine secretion or any sign of systemic toxicity. This aligns with previous studies that showed that low-avidity CAR T cells can lead to better anti-tumor effects [39] but includes the novel concept that even with similar affinities, we can obtain improved function by increasing the off-rates. Therefore, not only does h1218-CART19 have the potential to recognize lymphoma and leukemia characterized by FMC63 epitope loss, but it also shows increased anti-tumor activity when compared to FMC63-based CAR19 therapy due to reduced AICD.

Based on these preclinical results, we started a first-in-human multi-center phase I clinical trial to test its safety and feasibility in patients with relapsed or refractory NHL. In the phase I portion of the trial, 12 patients received 3 increasing doses of AT101 upon lymphodepletion. In patients receiving an active dose of AT101 (DL-2 and DL-3), the ORR was 100% and the CRR was 100%. Toxicities were manageable, with one case of severe CRS out of six patients treated at the highest dose levels and no severe ICANS at these doses. ICANS onset seemed to be slightly delayed as compared to current FDA-approved products, possibly due to the AT101 functional characteristics, although the numbers are too low for proper conclusions. Interestingly, AT101 was efficacious against multiple NHL types, particularly DLBCL and FL. Focusing on these common NHL, AT101 compares favorably with the known response rates of the FDA-approved axicabtagene ciloleucel, lisocabtagene maraleucel, and tisagenleceucel [33, 44], although the number of patients is limited in this early phase trial. It is promising that the expansion and persistence of AT101 were strong in these patients, confirming the preclinical results that suggest the low avidity of this CAR protects T cells from AICD and endows them with progressive proliferation over 30 days and extended persistence. Interestingly, we found that in all patients, key inflammatory cytokines, particularly the sFas ligand, granzyme A, and perforin, were elevated after CAR T infusion. We did not find a critical elevation of IL-6, IL-1, GM-CSF, TNF, or IFNγ in treated patients or those experiencing toxicity. This finding suggests that low avidity, fast on-/ off-rates, and different epitope recognition might lead to a different modulation of CAR T function and subsequent different cytokine profiles.

The cohort of patients treated with AT101 was heavily pretreated, including anti-CD19 antibodies, bispecific antibodies, and experimental NK cell therapy. Despite this, none of the patients with CR relapsed, although the follow-up period is still short. Although preclinical data suggest that AT101 can overcome some FMC63-resistance mechanisms such as CD19 epitope loss, this feature needs to be validated in prospective clinical trials. It is promising that, to date, no CD19-negative relapses have been observed in patients treated with AT101. This study also has limitations, such as the limited number of patients and the heterogeneity of patients, which are unavoidable characteristics of first-in-human early clinical trials. Moreover, the median vein-to-vein time was relatively long as compared to some of the currently approved CART19 products. While in the commercial settings is not uncommon to have 1–2 month of vein-to-vein time, we expect in the phase II trials to have a shorter duration of release tests which will reduce waiting time. Having defined the recommended phase II dose, the phase II portion of this trial will focus on the efficacy of AT101 in r/r DLBCL.

## Conclusion

In summary, we developed a CAR binder that could recognize an alternative membrane-proximal epitope of CD19 with fast on- and off-rates. These characteristics led to enhanced preclinical activity compared to FMC3-based CART19, owing to the different epitopes recognized, reduced AICD, and more gradual activation. We translated these findings into a first-in-human clinical trial that showed notable efficacy with 100% CR in DL-2 and -3 and manageable toxicity.

### Supplementary Information

**Additional file 1: Table S1.** Amino acid sequences of WT or modified CD19 extracellular domain. **Table S2.** CD19 CRISPR Short guide RNA sequence. **Table S3.** Clinical-grade AT101 product manufacturing information. **Table S4.** Patients' characteristics, response and toxicity. **Table S5.** ICANS clinical course and treatment. 

**Additional file 2: Figure S1.** h1218 antibody specificity characterization. A. Humanization of the chicken 1218 scFv by grafting the complementarity-determining regions (CDR) to human germline genes. Key framework region (FR) residues that were kept in the h1218 scFv were marked with *. B. Human HEK293 cell-based h1218 antibody binding (Retrogenix assay). Tested array included 2 control receptors and 16 potential hits derived from a previous binding screening using 5484 full-length human plasma membrane proteins and secreted proteins. h1218 showed specific binding to CD19 and to SUSD5, TMEM108 and GUCA2A on fixed (left) or live (right) cells. C. Confirmation of limited h1218 antibody binding ability to HEK293T WT cells or those overexpressed with TMEM-GFP or SUSD5-GFP by flow cytometry. D. Quantification of IFN-γ release from UTD or CART19 cells upon co-culture with WT HEK293T or those overexpressed with CD19, TMEM108, or SUSD5. E. Quantification of absorbance when various amounts of anti GUCA2A antibody, h1218 antibody or FMC63 antibody was added to GUCA2A secreted protein. All curves are represented as mean ± SEM. One-way ANOVA was performed with Tukey correction for multiple comparisons; **** *p* < 0.0001, *** *p* < 0.001, ** *p* < 0.01, * *p* < 0.05. **Figure S2.** FMC63- and h1218-CART19 cells demonstrated similar cytotoxic effects against four lymphoid cell lines at short term. A. Cytotoxicity on Raji, Pfeiffer, Toledo and Nalm6 after co-culture with UTD, FMC63-CART19 or h1218 CART19. B. Real-time kinetics analysis of Raji cell survival alone and in the presence of CART19-mediated cytotoxic activity over 48 hours (n=2 donors). Curves with error bars are represented as mean ± SEM. **Figure S3.** h1218-CART19 exhibited dose-dependent anti-tumor efficacy in xenograft models. A. 7 days after luciferase^+^ Raji cells intravenous injection (5x105 cells), vehicle, UTD and 4 doses (0.33x106, 1x106 and 3x106) of h1218-CART19 cells were administered. The tumor burden in mice bearing Raji was monitored over time. B. Tumor progression in low- and high-tumor-burden xenograft models. C. Replicate in vivo stress test experiment of FMC63- and h1218-CART19 cells against B-ALL Nalm6. (Left) Tumor burden in mice treated with UTD or CART19. (Right) Overall survival of the corresponding groups. **Figure S4.** AT101 phase I clinical trial design and patients. A. Schema of AT101 phase I clinical trial design. B. CONSORT diagram with patient screening, enrollment and treatment information. **Figure S5.** AT101 patient cell blood counts and ferritin levels. A. Patient-specific B cell count before and post AT101 infusion. B cell aplasia is defined as below 50 cells/μl. B. Patient-specific hemoglobin level over time. C. Patient-specific platelet level over time. D. Patient-specific ferritin level. **Figure S6.** AT101 patient response, progression-free survival, and overall survival. A. AT101 patient response 1 month (left) and 3 months (right) after infusion. B. Progression-free survival of patients treated with AT101 grouped based on response. C. Overall survival of patients treated with AT101 grouped based on response. **Figure S7.** AT101 patient serum cytokine levels. A. Level of perforin or sFas ligand quantified over time in individual patients. B. Level of MCP-4 or IP-10 quantified in individual patients. C. Level of IL-8 quantified in individual patients. D. Level of serum amyloid A quantified in individual patients.

## Data Availability

All requests for raw and analyzed materials are promptly reviewed by the University of Pennsylvania to determine whether they are subject to intellectual property or confidentiality obligations. Any data and materials that can be shared are released via a material-transfer agreement. Other data generated in this study are available from the corresponding authors upon reasonable request.

## Abbreviations

AICD: Activation-induced cell death
AE: Adverse event
ASTC: Autologous stem cell transplantation
ATCC: The American Type Culture Collection
(B-)ALL: (B-cell) acute lymphoblastic lymphoma
BLI: Bioluminescence intensity
CAR: Chimeric-antigen receptor
CBG: Click beetle green
CDR: Complementarity-determining regions
CR: Complete response
CRS: Cytokine release syndrome
CHOP: Cyclophosphamide, doxorubicin, vincristine
DL: Dose level
DLBCL: Diffuse large B-cell lymphoma
ECD: Extracellular domain
ESHAP: Etoposide, cisplatin, cytarabine, methylprednisolone
E:T: Effector: target
GFP: Green fluorescent protein
IACUC: Institutional Animal Care and Use Committee
IFN: Interferon
LD: Lymphodepletion
LDH: Lactate dehydrogenase
MFDS: Ministry of Food and Drug Safety
MFI: Mean fluorescence intensity
MTD: Maximum tolerated dose
NHL: Non-Hodgkin’s lymphoma
NSG: NOD-SCID gamma chain deficient
OR: Overall response
OS: Overall survival
PD: Progressive disease
PFD: Progression-free survival
PR: Partial response
qPCR: Quantitative polymerase chain reaction
RP2D: Recommended phase II dose
SD: Stable disease
SPD: Sum of the product of the diameters
STR: Short tandem repeat
UTD: Untransduced
