# Supplementary material for: Safety and efficacy of a novel anti-CD19 chimeric antigen receptor T cell product targeting a membrane-proximal domain of CD19 with fast on- and off-rates against non-Hodgkin lymphoma: a first-in-human study
Source: Mol Cancer. 2023 Dec 9;22:200. doi: 10.1186/s12943-023-01886-9 (PMC10709913; doi:10.1186/s12943-023-01886-9)
Supplement: Supplementary file 1 — Additional file 1: Table S1. Amino acid sequences of WT or modified CD19 extracellular domain. Table S2. CD19 CRISPR Short guide RNA sequence. Table S3. Clinical-grade AT101 product manufacturing information. Table S4. Patients' characteristics, response and toxicity. Table S5. ICANS clinical course and treatment. [file 12943_2023_1886_MOESM1_ESM.docx]

**SUPPLEMENTARY TABLES**

**Table S1. Amino acid sequences of WT or modified CD19 extracellular domain**

| **Construct** | **Sequence** |
| --- | --- |
| huCD19 | MPPPRLLFFLLFLTPMEVRPEEPLVVKVEEGDNAVLQCLKGTSDGPTQQLTWSRESPLKPFLKLSLGLPGLGIHMRPLAIWLFIFNVSQQMGGFYLCQPGPPSEKAWQPGWTVNVEGSGELFRWNVSDLGGLGCGLKNRSSEGPSSPSGKLMSPKLYVWAKDRPEIWEGEPPCLPPRDSLNQSLSQDLTMAPGSTLWLSCGVPPDSVSRGPLSWTHVHPKGPKSLLSLELKDDRPARDMWVMETGLLLPRATAQDAGKYYCHRGNLTMSFHLEITARPVLWHWLLRTGGWKVSAVTLA |
| Chimeric CD19 (**C**HH) | MPPPCLLFFLLFLTPMEVR**PQEPLVVKVEEGDNAVLQCLEGTSDGPTQQLVWCRDSPFEPFLNLSLGLPGMGIRMGPLGIWLLIFNVSNQTGGFYLCQPGLPSEKAWQPGWTV**NVEGSGELFRWNVSDLGGLGCGLKNRSSEGPSSPSGKLMSPKLYVWAKDRPEIWEGEPPCLPPRDSLNQSLSQDLTMAPGSTLWLSCGVPPDSVSRGPLSWTHVHPKGPKSLLSLELKDDRPARDMWVMETGLLLPRATAQDAGKYYCHRGNLTMSFHLEITARPVLWHWLLRTGGWKVSAVTLA |
| Chimeric CD19 (H**C**H) | MPPPRLLFFLLFLTPMEVRPEEPLVVKVEEGDNAVLQCLKGTSDGPTQQLTWSRESPLKPFLKLSLGLPGLGIHMRPLAIWLFIFNVSQQMGGFYLCQPGPPSEKAWQPGWTV**SVEGSGELFRWNVSDLGGLGCGLKNRSSEGPSSPSGKLNSSQLYVWAKDRPEMWEGEPVCGP**PRDSLNQSLSQDLTMAPGSTLWLSCGVPPDSVSRGPLSWTHVHPKGPKSLLSLELKDDRPARDMWVMETGLLLPRATAQDAGKYYCHRGNLTMSFHLEITARPVLWHWLLRTGGWKVSAVTLA |
| Chimeric CD19 (HH**C**) | MPPPRLLFFLLFLTPMEVRPEEPLVVKVEEGDNAVLQCLKGTSDGPTQQLTWSRESPLKPFLKLSLGLPGLGIHMRPLAIWLFIFNVSQQMGGFYLCQPGPPSEKAWQPGWTVNVEGSGELFRWNVSDLGGLGCGLKNRSSEGPSSPSGKLMSPKLYVWAKDRPEIWEGEPPCLP**PRDSLNQSLSQDLTMAPGSTLWLSCGVPPDSVSRGPLSWTHVRPKGPKSSLLSLELKDDRPDRDMWVVDTGLLLTRATAQDAGKYYCHRGNWTKSFYLEITAR**PVLWHWLLRTGGWKVSAVTLA |
| MutCD19 (E21Q) | MPPPRLLFFLLFLTPMEVRP**Q**EPLVVKVEEGDNAVLQCLKGTSDGPTQQLTWSRESPLKPFLKLSLGLPGLGIHMRPLAIWLFIFNVSQQMGGFYLCQPGPPSEKAWQPGWTVNVEGSGELFRWNVSDLGGLGCGLKNRSSEGPSSPSGKLMSPKLYVWAKDRPEIWEGEPPCLPPRDSLNQSLSQDLTMAPGSTLWLSCGVPPDSVSRGPLSWTHVHPKGPKSLLSLELKDDRPARDMWVMETGLLLPRATAQDAGKYYCHRGNLTMSFHLEITARPVLWHWLLRTGGWKVSAVTLA |
| MutCD19 (K40E) | MPPPRLLFFLLFLTPMEVRPEEPLVVKVEEGDNAVLQCL**E**GTSDGPTQQLTWSRESPLKPFLKLSLGLPGLGIHMRPLAIWLFIFNVSQQMGGFYLCQPGPPSEKAWQPGWTVNVEGSGELFRWNVSDLGGLGCGLKNRSSEGPSSPSGKLMSPKLYVWAKDRPEIWEGEPPCLPPRDSLNQSLSQDLTMAPGSTLWLSCGVPPDSVSRGPLSWTHVHPKGPKSLLSLELKDDRPARDMWVMETGLLLPRATAQDAGKYYCHRGNLTMSFHLEITARPVLWHWLLRTGGWKVSAVTLA |
| MutCD19 (T51V) | MPPPRLLFFLLFLTPMEVRPEEPLVVKVEEGDNAVLQCLKGTSDGPTQQL**V**WSRESPLKPFLKLSLGLPGLGIHMRPLAIWLFIFNVSQQMGGFYLCQPGPPSEKAWQPGWTVNVEGSGELFRWNVSDLGGLGCGLKNRSSEGPSSPSGKLMSPKLYVWAKDRPEIWEGEPPCLPPRDSLNQSLSQDLTMAPGSTLWLSCGVPPDSVSRGPLSWTHVHPKGPKSLLSLELKDDRPARDMWVMETGLLLPRATAQDAGKYYCHRGNLTMSFHLEITARPVLWHWLLRTGGWKVSAVTLA |
| MutCD19 (S53C) | MPPPRLLFFLLFLTPMEVRPEEPLVVKVEEGDNAVLQCLKGTSDGPTQQLTW**C**RESPLKPFLKLSLGLPGLGIHMRPLAIWLFIFNVSQQMGGFYLCQPGPPSEKAWQPGWTVNVEGSGELFRWNVSDLGGLGCGLKNRSSEGPSSPSGKLMSPKLYVWAKDRPEIWEGEPPCLPPRDSLNQSLSQDLTMAPGSTLWLSCGVPPDSVSRGPLSWTHVHPKGPKSLLSLELKDDRPARDMWVMETGLLLPRATAQDAGKYYCHRGNLTMSFHLEITARPVLWHWLLRTGGWKVSAVTLA |
| MutCD19 (E55D) | MPPPRLLFFLLFLTPMEVRPEEPLVVKVEEGDNAVLQCLKGTSDGPTQQLTWSR**D**SPLKPFLKLSLGLPGLGIHMRPLAIWLFIFNVSQQMGGFYLCQPGPPSEKAWQPGWTVNVEGSGELFRWNVSDLGGLGCGLKNRSSEGPSSPSGKLMSPKLYVWAKDRPEIWEGEPPCLPPRDSLNQSLSQDLTMAPGSTLWLSCGVPPDSVSRGPLSWTHVHPKGPKSLLSLELKDDRPARDMWVMETGLLLPRATAQDAGKYYCHRGNLTMSFHLEITARPVLWHWLLRTGGWKVSAVTLA |
| MutCD19 (L58F) | MPPPRLLFFLLFLTPMEVRPEEPLVVKVEEGDNAVLQCLKGTSDGPTQQLTWSRESP**F**KPFLKLSLGLPGLGIHMRPLAIWLFIFNVSQQMGGFYLCQPGPPSEKAWQPGWTVNVEGSGELFRWNVSDLGGLGCGLKNRSSEGPSSPSGKLMSPKLYVWAKDRPEIWEGEPPCLPPRDSLNQSLSQDLTMAPGSTLWLSCGVPPDSVSRGPLSWTHVHPKGPKSLLSLELKDDRPARDMWVMETGLLLPRATAQDAGKYYCHRGNLTMSFHLEITARPVLWHWLLRTGGWKVSAVTLA |
| MutCD19 (K59E) | MPPPRLLFFLLFLTPMEVRPEEPLVVKVEEGDNAVLQCLKGTSDGPTQQLTWSRESPL**E**PFLKLSLGLPGLGIHMRPLAIWLFIFNVSQQMGGFYLCQPGPPSEKAWQPGWTVNVEGSGELFRWNVSDLGGLGCGLKNRSSEGPSSPSGKLMSPKLYVWAKDRPEIWEGEPPCLPPRDSLNQSLSQDLTMAPGSTLWLSCGVPPDSVSRGPLSWTHVHPKGPKSLLSLELKDDRPARDMWVMETGLLLPRATAQDAGKYYCHRGNLTMSFHLEITARPVLWHWLLRTGGWKVSAVTLA |
| MutCD19 (K63N) | MPPPRLLFFLLFLTPMEVRPEEPLVVKVEEGDNAVLQCLKGTSDGPTQQLTWSRESPLKPFL**N**LSLGLPGLGIHMRPLAIWLFIFNVSQQMGGFYLCQPGPPSEKAWQPGWTVNVEGSGELFRWNVSDLGGLGCGLKNRSSEGPSSPSGKLMSPKLYVWAKDRPEIWEGEPPCLPPRDSLNQSLSQDLTMAPGSTLWLSCGVPPDSVSRGPLSWTHVHPKGPKSLLSLELKDDRPARDMWVMETGLLLPRATAQDAGKYYCHRGNLTMSFHLEITARPVLWHWLLRTGGWKVSAVTLA |
| MutCD19 (L71M) | MPPPRLLFFLLFLTPMEVRPEEPLVVKVEEGDNAVLQCLKGTSDGPTQQLTWSRESPLKPFLKLSLGLPG**M**GIHMRPLAIWLFIFNVSQQMGGFYLCQPGPPSEKAWQPGWTVNVEGSGELFRWNVSDLGGLGCGLKNRSSEGPSSPSGKLMSPKLYVWAKDRPEIWEGEPPCLPPRDSLNQSLSQDLTMAPGSTLWLSCGVPPDSVSRGPLSWTHVHPKGPKSLLSLELKDDRPARDMWVMETGLLLPRATAQDAGKYYCHRGNLTMSFHLEITARPVLWHWLLRTGGWKVSAVTLA |
| MutCD19 (R76G) | MPPPRLLFFLLFLTPMEVRPEEPLVVKVEEGDNAVLQCLKGTSDGPTQQLTWSRESPLKPFLKLSLGLPGLGIHM**G**PLAIWLFIFNVSQQMGGFYLCQPGPPSEKAWQPGWTVNVEGSGELFRWNVSDLGGLGCGLKNRSSEGPSSPSGKLMSPKLYVWAKDRPEIWEGEPPCLPPRDSLNQSLSQDLTMAPGSTLWLSCGVPPDSVSRGPLSWTHVHPKGPKSLLSLELKDDRPARDMWVMETGLLLPRATAQDAGKYYCHRGNLTMSFHLEITARPVLWHWLLRTGGWKVSAVTLA |
| MutCD19 (A79G) | MPPPRLLFFLLFLTPMEVRPEEPLVVKVEEGDNAVLQCLKGTSDGPTQQLTWSRESPLKPFLKLSLGLPGLGIHMRPL**G**IWLFIFNVSQQMGGFYLCQPGPPSEKAWQPGWTVNVEGSGELFRWNVSDLGGLGCGLKNRSSEGPSSPSGKLMSPKLYVWAKDRPEIWEGEPPCLPPRDSLNQSLSQDLTMAPGSTLWLSCGVPPDSVSRGPLSWTHVHPKGPKSLLSLELKDDRPARDMWVMETGLLLPRATAQDAGKYYCHRGNLTMSFHLEITARPVLWHWLLRTGGWKVSAVTLA |
| MutCD19 (F83L) | MPPPRLLFFLLFLTPMEVRPEEPLVVKVEEGDNAVLQCLKGTSDGPTQQLTWSRESPLKPFLKLSLGLPGLGIHMRPLAIWL**L**IFNVSQQMGGFYLCQPGPPSEKAWQPGWTVNVEGSGELFRWNVSDLGGLGCGLKNRSSEGPSSPSGKLMSPKLYVWAKDRPEIWEGEPPCLPPRDSLNQSLSQDLTMAPGSTLWLSCGVPPDSVSRGPLSWTHVHPKGPKSLLSLELKDDRPARDMWVMETGLLLPRATAQDAGKYYCHRGNLTMSFHLEITARPVLWHWLLRTGGWKVSAVTLA |
| MutCD19 (H218R/KSS) | MPPPRLLFFLLFLTPMEVRPEEPLVVKVEEGDNAVLQCLKGTSDGPTQQLTWSRESPLKPFLKLSLGLPGLGIHMRPLAIWLFIFNVSQQMGGFYLCQPGPPSEKAWQPGWTVNVEGSGELFRWNVSDLGGLGCGLKNRSSEGPSSPSGKLMSPKLYVWAKDRPEIWEGEPPCLPPRDSLNQSLSQDLTMAPGSTLWLSCGVPPDSVSRGPLSWTHV**R**PKGPK**S**SLLSLELKDDRPARDMWVMETGLLLPRATAQDAGKYYCHRGNLTMSFHLEITARPVLWHWLLRTGGWKVSAVTLA |

**mutated human CD19 at position 224 (serine addition, KS 🡪 KSS) [27]*

**Table S2: CD19 CRISPR Short guide RNA sequence**

| **Gene name** | **Sequence** |
| --- | --- |
| CD19 | TCGCCCGGCCAGAGATATGT |

| **Patient #** | **1** | **3** | **5** | **6** | **7** | **8** | **9** | **10** | **11** | **12** | **13** | **14** |
| --- | --- | --- | --- | --- | --- | --- | --- | --- | --- | --- | --- | --- |
| **Population doubling (Day11)** | 6.0 | 5.6 | 5.6 | 5.4 | 5.5 | 5.4 | 5.7 | 5.6 | 5.8 | 5.2 | 5.6 | 5.6 |
| **Fold change (Day11)** | 62 | 53 | 47 | 44 | 51 | 46 | 60 | 57 | 57 | 38 | 45 | 55 |
| **Leukapheresis CD4/CD8 ratio** | 2.1 | 0.7 | 0.5 | 1.8 | 0.4 | 0.5 | 1.4 | 0.6 | 1.8 | 0.1 | 0.3 | 1.7 |
| **Final Product CD4/CD8 ratio** | 1.7 | 0.5 | 0.1 | 1.2 | 0.5 | 0.1 | 1.2 | 0.8 | 1.1 | 0.1 | 0.3 | 0.6 |
| **CD45+CD3+ (%)** | 94 | 88 | 91 | 94 | 93 | 90 | 95 | 92 | 92 | 93 | 86 | 93 |
| **CD45+CD3-CD19+ (%)** | 0 | 0 | 0 | 0 | 0 | 0 | 0 | 0 | 0 | 0 | 0 | 0 |
| **CAR expression (%)** | 46 | 46 | 25 | 47 | 35 | 37 | 45 | 50 | 50 | 41 | 51 | 57 |
|  |  |  |  |  |  |  |  |  |  |  |  |  |
| **Leukapheresis to Product formulation (Day)** | 11 | 11 | 11 | 11 | 11 | 11 | 11 | 11 | 11 | 11 | 15 | 7 |
| **Product formulation to Final QC test (Day)** | 35 | 34 | 33 | 33 | 36 | 35 | 34 | 34 | 36 | 36 | 37 | 41 |
| **Final QC to Product release (Day)** | 3 | 3 | 49 | 3 | 5 | 12 | 7 | 10 | 1 | 9 | 7 | 8 |
| **Product release to infusion (Day)** | 1 | 1 | 4 | 1 | 1 | 7 | 1 | 4 | 4 | 4 | 4 | 3 |
| **Vein to vein (Day)** | 50 | 49 | 97 | 48 | 53 | 65 | 53 | 59 | 52 | 60 | 63 | 59 |
| **Cause of delay** | - | - | COVID19 | - | - | - | - | - | - | - | - | - |

**Table S3. Clinical-grade AT101 product manufacturing information**

**Table S4: Patients' Characteristics, Response and Toxicity**

| **Patient #** | **1** | **3** | **5** | **6** | **7** | **8** | **9** | **10** | **11** | **12** | **13** | **14** |
| --- | --- | --- | --- | --- | --- | --- | --- | --- | --- | --- | --- | --- |
| **Registration number** | R101001 | R10  100  3 | R101005 | R101006 | R101007 | R102001 | R101008 | R102002 | R102003 | R102004 | R103001 | R104001 |
| ***Characteristics*** |  |  |  |  |  |  |  |  |  |  |  |  |
| **Dose Level** | 1 | 1 | 1 | 1 | 1 | 1 | 2 | 2 | 2 | 3 | 3 | 3 |
| **Age, years** | 39 | 68 | 72 | 84 | 49 | 63 | 54 | 72 | 53 | 77 | 62 | 59 |
| **Sex** | F | F | M | F | M | M | F | M | F | F | M | F |
| **ECOG performance status** | 0 | 0 | 1 | 1 | 1 | 1 | 1 | 1 | 1 | 1 | 0 | 1 |
| **Histology** | FL | DLBCL | MZL | DLBCL | FL | MCL | DLBCL | FL | DLBCL | DLBCL | DLBCL | DLBCL |
| **Number of prior lines of therapy** | 3 | 2 | 2 | 4 | 8 | 3 | 2 | 6 | 2 | 3 | 2 | 3 |
| **Previous lines of therapy** | 1. RCVP/ rituximab maintenance  2. Mosunetuzumab  3. Duvelisib | 1. R-CHOP + lenalidomide  2.Bendamustine, Tafasitamab | 1. RCVP  2. RCHOP | 1.CHOP  2. RCHOP  3. GDP  4. Epcoritamab | 1. RCHOP/ Rituximab maintenance  2. ICE-D/BuCyE-ASCT  3. B-NHL – polychemo  4. CD20 radioimmunotherapy  5. FC-lite  6. Bendamustine  7. Retuxima-Bendamustine/  8. Lenalidomide,Rituximab | 1. RCHOP  2. Rituximab,Bendamustine  3. DHAP | 1. RCHOP  2. ICE-D/BeEAM-ASCT | 1. RCVP  2. CEOP  3. MG4101-Rituximab  4. CEOP  5. Rituximab,Bendamustine  6. RCHOP | 1. RCHOP  2. ICE-D/BuCyE-ASCT | 1. RCHOP  2. Rituximab,Bendamustine  3. ICE-D | 1. RCHOP  2. RCHOP | 1. RCHOP  2. RDHAP  3. Odronextamab |
| **Prior ASCT** | - | - | - | - | Y | Y | Y | - | Y | - | - | Y |
| **Prior CD20/CD3 bispecific ab** | Y  (Mosunetuzumab) | - | - | Y  (Epcoritamab) | - | - | - | - | - | - | - | Y  (Odronextamab) |
| **Prior CD19-directed therapy** | - | Y  (Tafasitamab) | - | - | - | - | - | - | - | - | - | - |
| **Prior Cellular Therapy** | - | - | - | - | - | - |  | Y  (MG4101) | - | - | - | - |
| **Baseline SPD (mm^2^)** | 1,067 | 1,002 | 11,836 | 2,796 | -* | 2,483 | 190 | 6,128 | 300 | -* | 1,015 | 617.55 |
| **Bulky disease** | - | - | Y | - | - | - | - | - | - | - | - | - |
| **Bridging therapy** | - | - | - | - | - | - | Y  (CHOP) | - | - | Y  (ESHAP) | - | - |
| **LDH pre-LD (IU/L)** | 141 | 293 | 266 | 234 | 222 | 338 | 199 | 198 | 185 | 599 | 242 | 339 |
| **Elevated LDH** | - | Y | Y | - | - | Y | - | - | - | Y | Y | - |
| ***Response*** |  |  |  |  |  |  |  |  |  |  |  |  |
| **Best overall response** | CR | CR | PR | PD | CR | PR | CR | CR | CR | CR | CR | CR |
| ***Toxicity*** |  |  |  |  |  |  |  |  |  |  |  |  |
| **CRS grade** | - | - | - | - | - | - | - | - | 1 | 1 | 1 | 3 |
| **ICANS grade** | 4 | - | - | - | - | - | - | - | 1 | - | - | 2 |
| **Neutropenia grade** | 3 | 2 | - | 4 | 4 | 4 | 4 | - | - | 4 | 2 | 4 |
| **Anemia grade** | 3 | - | - | 2 | 3 | 3 | - | - | - | 3 | - | - |
| **Thrombocytopenia grade** | - | - | - | - | - | 3 | - | - | - | 4 | - | 2 |
| **Diarrhea grade** | - | - | - | - | - | - | - | 2 | - | 3 | 1 | - |
| **Hypokalemia grade** | - | - | - | - | - | - | - | - | - | 3 | - | - |
| **Pruritus grade** | - | - | - | 1 | - | - | 1 | - | 2 | 2 | - | - |
| **Sepsis grade** | - | - | - | - | - | - | - | - | - | 5 | - | - |
| **Pain grade** | - | - | - | 1 | - | - | 1 | - | 2 | 2 | - | - |
| **Anorexia grade** | - | - | - | - | - | 1 | - | - | - | 3 | - | 1 |
| **Infection grade** | 1 | - | - | 3 | - | - | - | - | - | 3 | - | - |
| **Insomnia grade** | - | - | - | - | 1 | 1 | - | - | - | - | - | - |
| **Hypotension grade** | - | - | - | - | 2 | - | - | - | 2 | - | - | - |
| **Pneumonia grade** | - | - | - | 3 | - | - | - | - | - | - | - | - |
| **Fever grade** | - | - | - | - | - | - | - | - | 1 | 1 | 2 | 1 |

** No measurable tumor lesion.*

*Abbreviations: DLBCL, diffuse large B cell lymphoma; FL, follicular lymphoma; MCL, mantle cell lymphoma; MZL, marginal zone lymphoma; NA, Not analyzed; ASCT, autologous stem cell transplantation; SPD, sum of the product of the diameters.*

*Bulky disease defined as any mass >7 cm.*

*Institution differences in serum LDH normal range:*

*Patient 1, Patient 3, Patient 5, Patient 6, Patient 7, and Patient 9: 120-250 IU/L*

*Patient 8, Patient 10, Patient 11, Patient 12: 106-230 IU/L*

*Patient 13: 100-200 IU/L*

*Patient 14: 140-480 IU/L.*

**Table S5. ICANS clinical course and treatment**

| **Hospital** | **Patient #** | **Grade** | **Symptoms** | **Treatment** | **Onset (Day)** | **Duration (Days)** |
| --- | --- | --- | --- | --- | --- | --- |
| Asan Medical Center | 1 | 4 | Tremor Seizures  Severely altered mental status | Levetiracetam, Lacosamide, Acyclovir, Lorazepam, Methylprednisolone, Propofol, Etomidate, Succinylcholine chloride, Fentanyl citrate, Mannitol, Furosemide, | 12 | 6 |
| Ulsan University Hospital | 11 | 1 | Tremors | Dexamethasone | 12 | 2 |
| Dong-A University Hospital | 14 | 2 | Delirium | Dexamethasone, Levetiracetam | 8 | 3 |
