# Supplementary material for: Safety and efficacy of a novel anti-CD19 chimeric antigen receptor T cell product targeting a membrane-proximal domain of CD19 with fast on- and off-rates against non-Hodgkin lymphoma: a first-in-human study
Source: Mol Cancer. 2023 Dec 9;22:200. doi: 10.1186/s12943-023-01886-9 (PMC10709913; doi:10.1186/s12943-023-01886-9)
Supplement: Supplementary file 2 — Additional file 2: Figure S1. h1218 antibody specificity characterization. A. Humanization of the chicken 1218 scFv by grafting the complementarity-determining regions (CDR) to human germline genes. Key framework region (FR) residues that were kept in the h1218 scFv were marked with *. B. Human HEK293 cell-based h1218 antibody binding (Retrogenix assay). Tested array included 2 control receptors and 16 potential hits derived from a previous binding screening using 5484 full-length human plasma membrane proteins and secreted proteins. h1218 showed specific binding to CD19 and to SUSD5, TMEM108 and GUCA2A on fixed (left) or live (right) cells. C. Confirmation of limited h1218 antibody binding ability to HEK293T WT cells or those overexpressed with TMEM-GFP or SUSD5-GFP by flow cytometry. D. Quantification of IFN-γ release from UTD or CART19 cells upon co-culture with WT HEK293T or those overexpressed with CD19, TMEM108, or SUSD5. E. Quantification of absorbance when various amounts of anti GUCA2A antibody, h1218 antibody or FMC63 antibody was added to GUCA2A secreted protein. All curves are represented as mean ± SEM. One-way ANOVA was performed with Tukey correction for multiple comparisons; **** p < 0.0001, *** p < 0.001, ** p < 0.01, * p < 0.05. Figure S2. FMC63- and h1218-CART19 cells demonstrated similar cytotoxic effects against four lymphoid cell lines at short term. A. Cytotoxicity on Raji, Pfeiffer, Toledo and Nalm6 after co-culture with UTD, FMC63-CART19 or h1218 CART19. B. Real-time kinetics analysis of Raji cell survival alone and in the presence of CART19-mediated cytotoxic activity over 48 hours (n=2 donors). Curves with error bars are represented as mean ± SEM. Figure S3. h1218-CART19 exhibited dose-dependent anti-tumor efficacy in xenograft models. A. 7 days after luciferase+ Raji cells intravenous injection (5x10^5^ cells), vehicle, UTD and 4 doses (0.33x10^6^, 1x10^6^ and 3x10^6^) of h1218-CART19 cells were administered. The tumor burden in mice bearing Raji [file 12943_2023_1886_MOESM2_ESM.pdf]

Figure S1

A

Chicken and humanized 1218 scFv sequence

V$_L$

	FR1	CDR1	FR2	CDR2	FR3	CDR3	FR4
opt. 1218	XXALTQPSSVSANPGETVKITC	SGGYSS----YYG	WYQQKSPGSAPVTLIY	ESNKRPS	DIPSRFSGSASGSTATLTITGVQVEDEAVYYC	GGLTPTHAGI	FGAGTTLTVL
			*		* *		
IGLV1-51*02 IGLJ2*01	QSVLTQPPSVSAAPGQKVTISC	SGSSSNIGNNYVS	WYQQL-PGTAPKLLIY	ENNKRPS	GIPDRFSGSKSGTSATLGITGLQTGDEADYYC	GTWDSSLSA VV	FGGGTKLTVL
IGLJ2*01	QSVLTQPPSVSAAPGQKVTISC	SGGYSS----YYG	WYQQL-PGTAPKTLIY	ESNKRPS	GIPDRFSGSASGSSATLGITGLQTGDEADYYC	GGLTPTHAGI	FGGGTKLTVL

V$_H$

	FR1	CDR1	FR2	CDR2	FR3	CDR3	FR4
opt.1218	AVTLDESGGGLQTPGGALSLVCKASGFTFS	SYDMG	WVRQAPGKGLEFVA	GIDDDGRYTSYGSAVDG	RATISRDNGQSTVRLQLNNLRAEDTATYYCTR	GNAGWIDA	WGHGTEVIVSS
			****		* *		
IGHV3-21*04 IGHJ5*01	EVQLVESGGGLVKPGGSLRLSCAASGFTFS	SYSMN	WVRQAPGKGLEWVS	SISSSSSYIYYADSVKG	RFTISRDNAKNSLYLQMNSLRAEDTAVYYCAR	AEYFQH	WGQGTLVTVSS
h1218	EVQLVESGGGLVQPGGSLRLSCAASGFTFS	SYDMG	WVRQAPGKGLEFVA	GIDDDGRYTSYGSAVDG	RATISRDNAKNTLYLQMNSLRAEDTAVYYCTR	GNAGWIDA	WGQGTLVTVSS

B

h1218 specificity assay (Retrogenix)

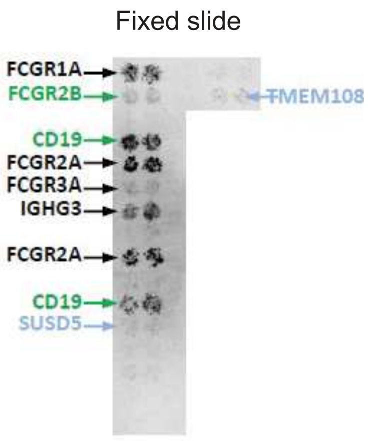

C

Reactivity of h1218 scFv against potential off-targets

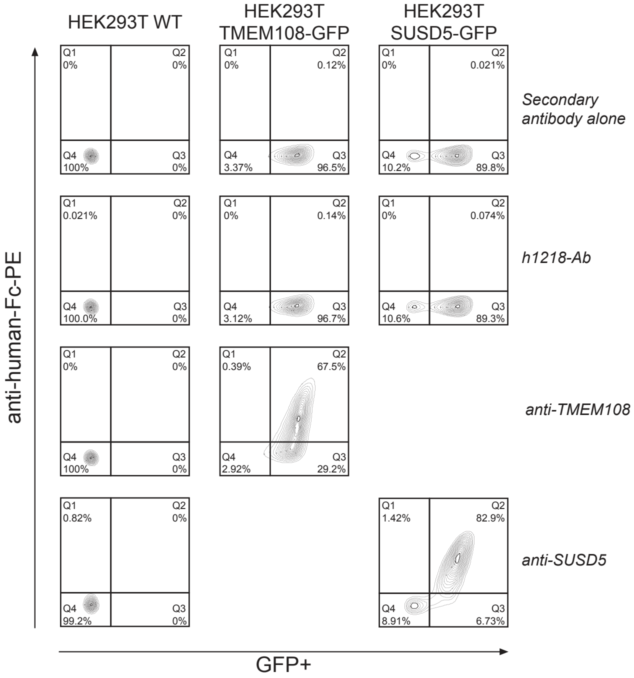

D

h1218 CART19 binding toTMEM108 or SUSD5 expressed in 293T cells

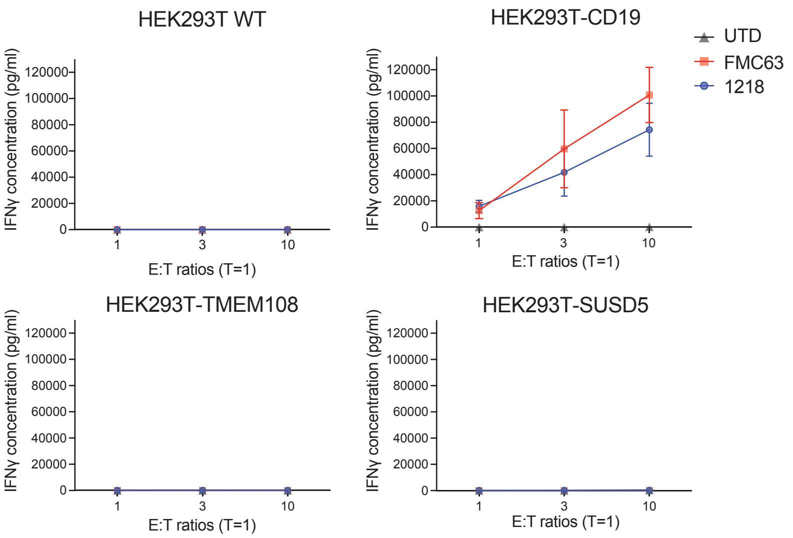

E

h1218 CART19 binding to secreted protein GUCA2A

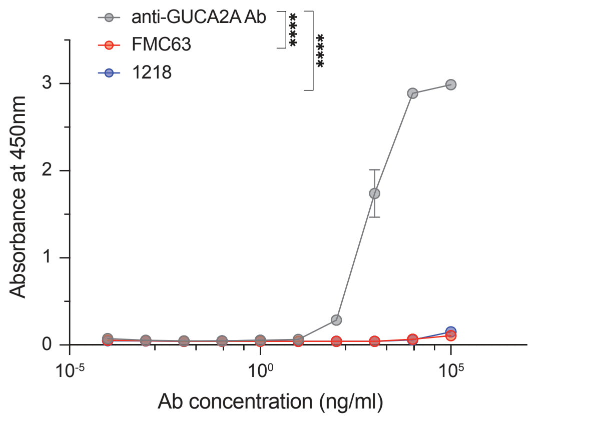

Figure S2

A **Short-term (72 hours) killing assay against different B cell lymphoma / leukemia**

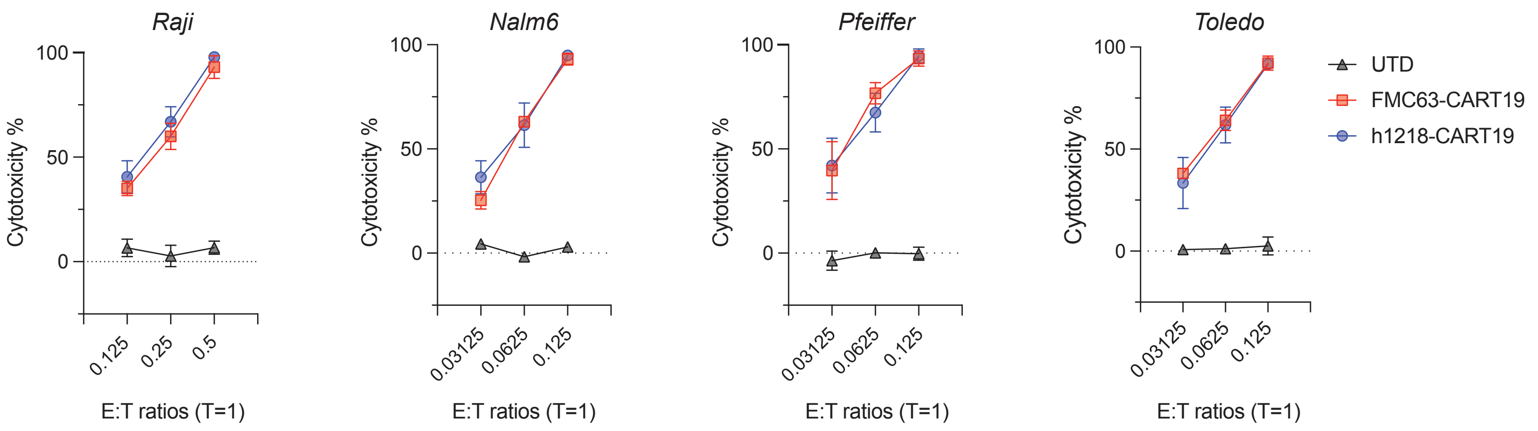

B **Short-term (48 hours) kinetic analysis using live cell imaging**

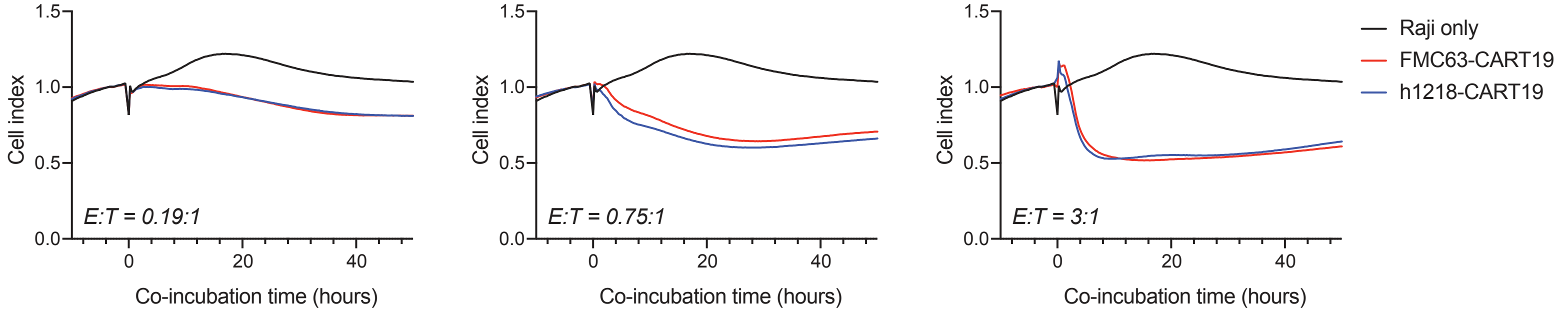

Figure S3

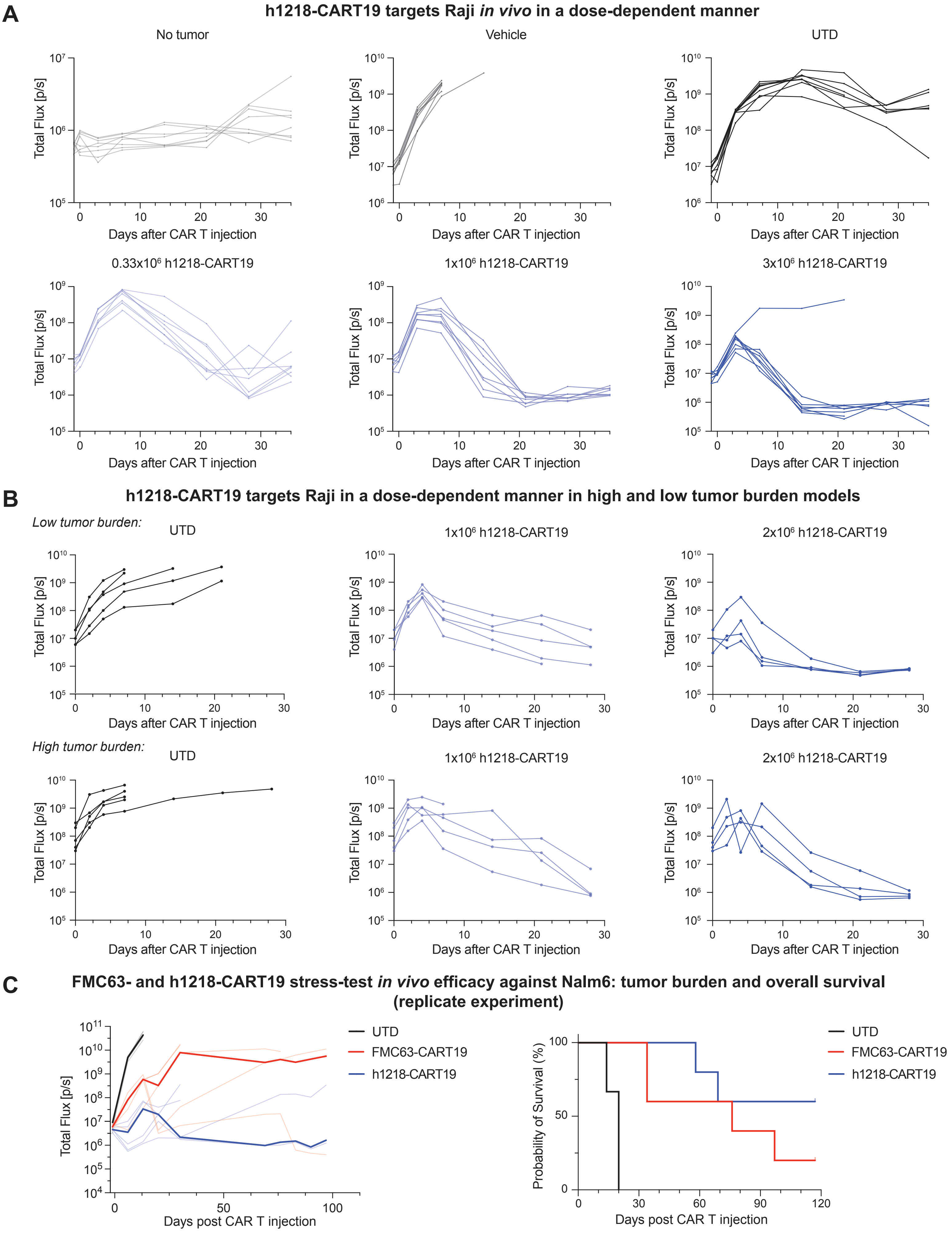
A
h1218-CART19 targets Raji in vivo in a dose-dependent manner
No tumor
Vehicle
UTD
0.33x10⁶ h1218-CART19
1x10⁶ h1218-CART19
3x10⁶ h1218-CART19
Total Flux [p/s]
Days after CAR T injection
B
h1218-CART19 targets Raji in a dose-dependent manner in high and low tumor burden models
Low tumor burden:
UTD
1x10⁶ h1218-CART19
2x10⁶ h1218-CART19
High tumor burden:
UTD
1x10⁶ h1218-CART19
2x10⁶ h1218-CART19
Total Flux [p/s]
Days after CAR T injection
C
FMC63- and h1218-CART19 stress-test in vivo efficacy against Nalm6: tumor burden and overall survival (replicate experiment)
UTD
FMC63-CART19
h1218-CART19
Total Flux [p/s]
Days post CAR T injection
Probability of Survival (%)
Days post CAR T injection

Figure S4

A

AT101 phase I clinical trial schema

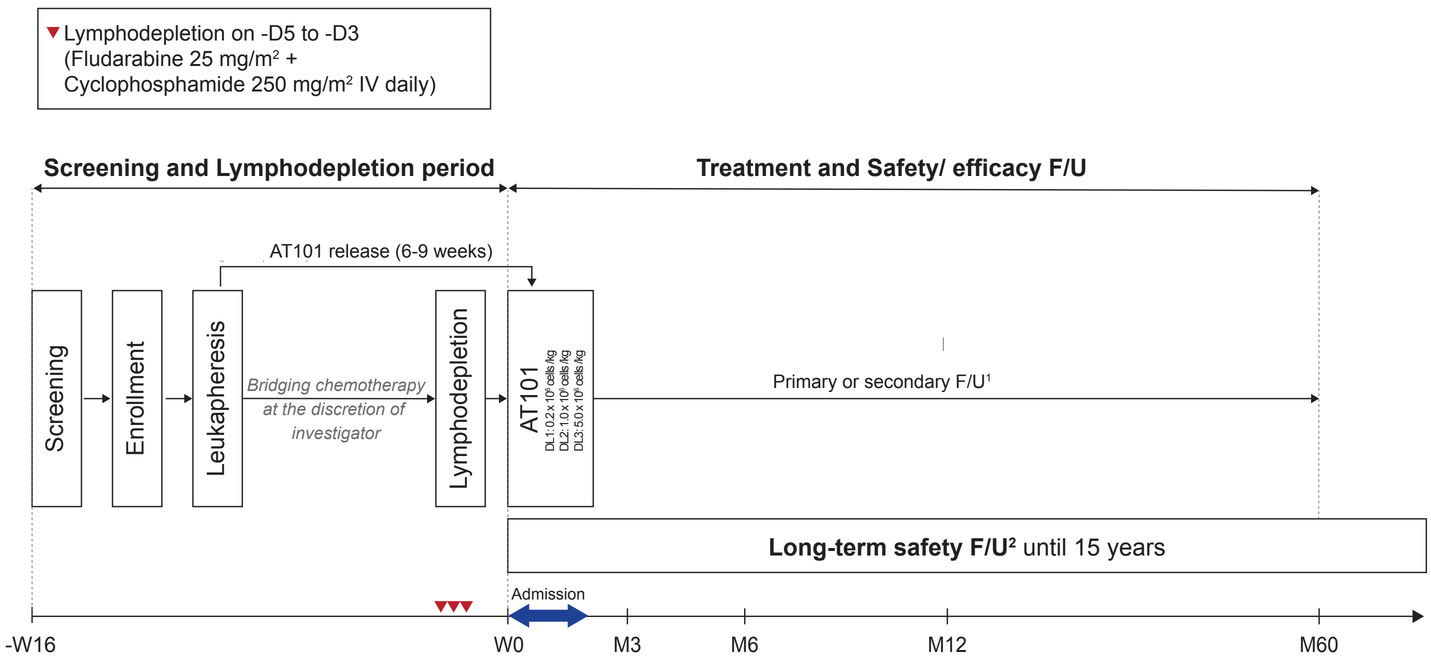

B

CONSORT diagram of AT101 phase I clinical trial: screening, enrollment, and treatment

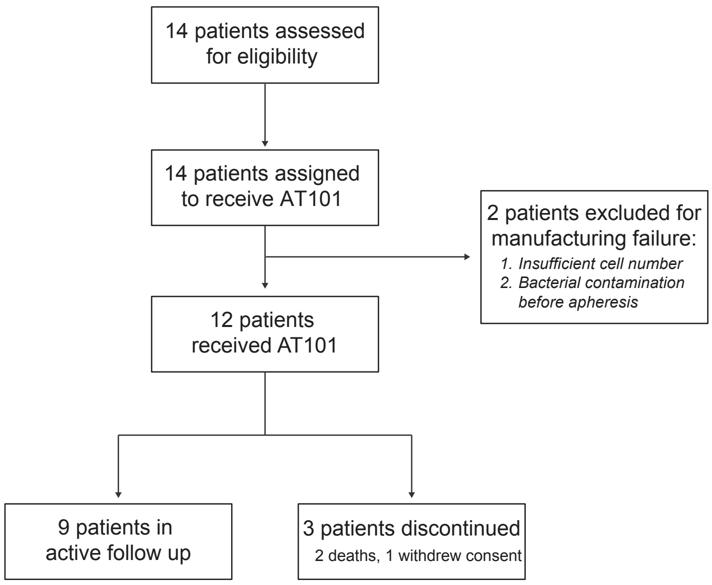

Figure S5

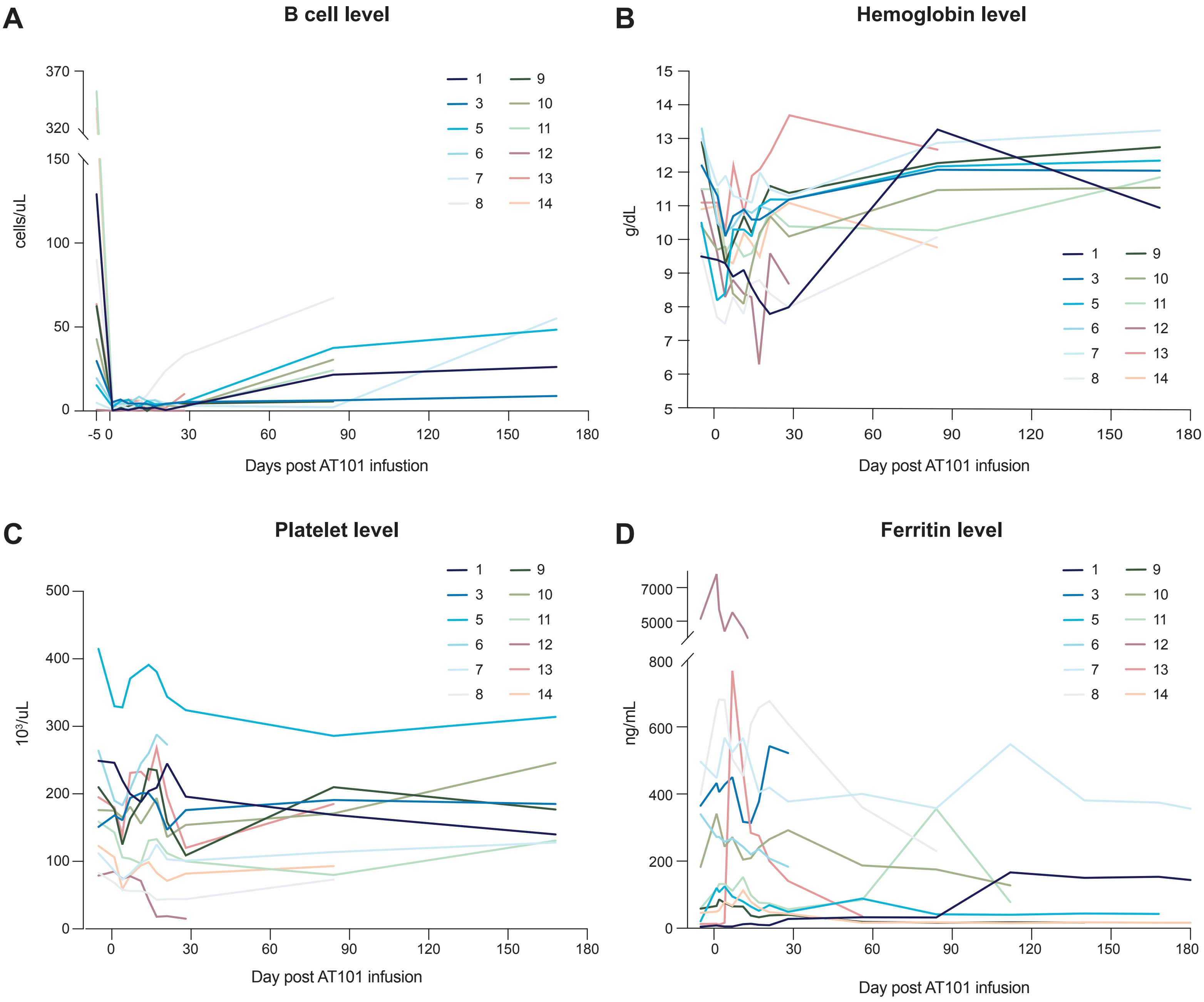

A
B cell level
370
320
150
100
50
0
cells/uL
-5 0
30
60
90
120
150
180
Days post AT101 infusion
1
3
5
6
7
8
9
10
11
12
13
14
B
Hemoglobin level
15
14
13
12
11
10
9
8
7
6
5
g/dL
0
30
60
90
120
150
180
Day post AT101 infusion
1
3
5
6
7
8
9
10
11
12
13
14
C
Platelet level
500
400
300
200
100
0
10^3/uL
0
30
60
90
120
150
180
Day post AT101 infusion
1
3
5
6
7
8
9
10
11
12
13
14
D
Ferritin level
7000
5000
800
600
400
200
0
ng/mL
0
30
60
90
120
150
180
Day post AT101 infusion
1
3
5
6
7
8
9
10
11
12
13
14

Figure S6

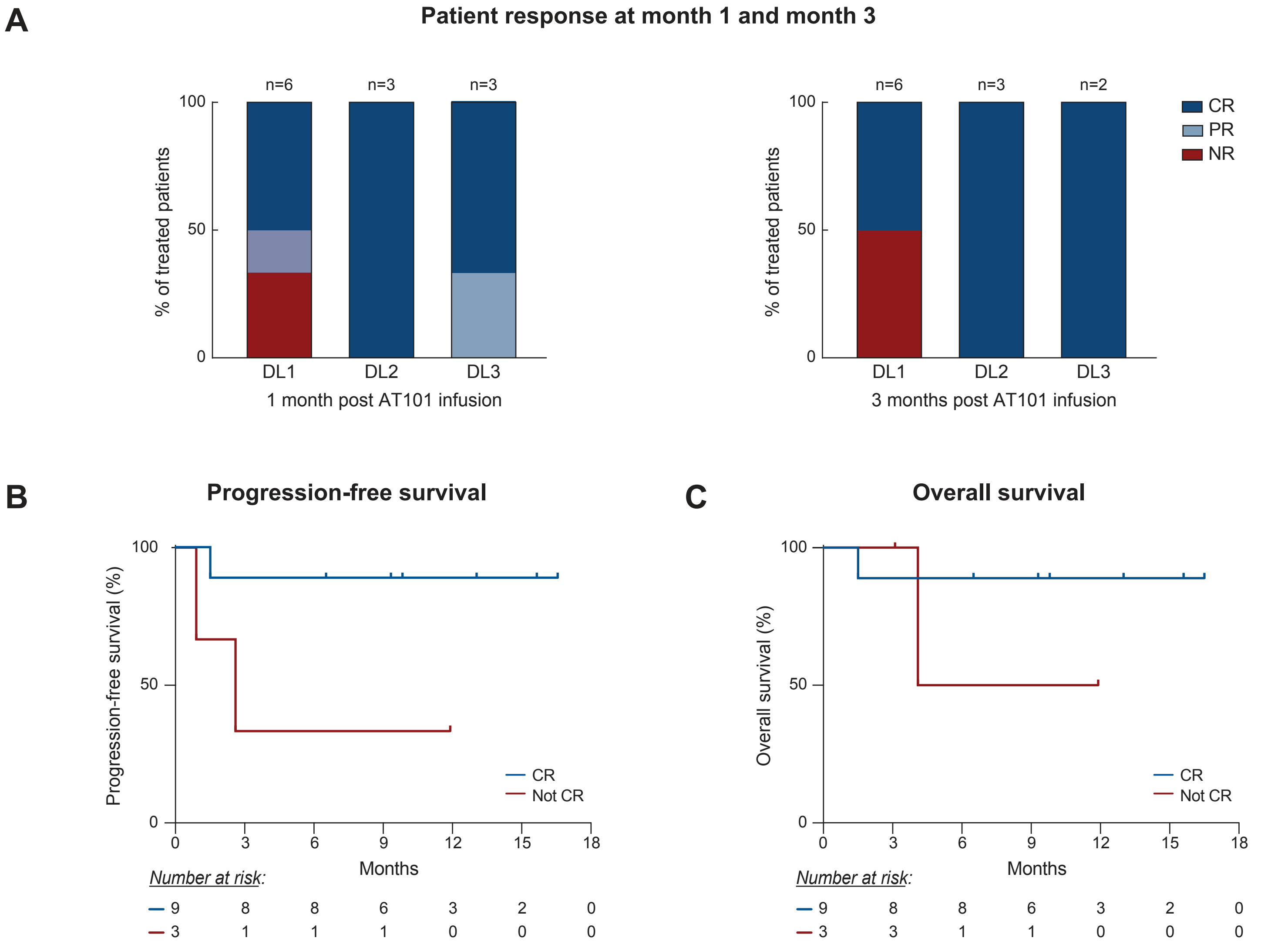
A
Patient response at month 1 and month 3
n=6
n=3
n=3
100
50
0
% of treated patients
DL1
DL2
DL3
1 month post AT101 infusion
n=6
n=3
n=2
3 months post AT101 infusion
CR
PR
NR
B
Progression-free survival
Progression-free survival (%)
Months
0
3
6
9
12
15
18
CR
Not CR
Number at risk:
9 8 8 6 3 2 0
3 1 1 1 0 0 0
C
Overall survival
Overall survival (%)
Months
CR
Not CR
Number at risk:
9 8 8 6 3 2 0
3 3 1 1 0 0 0

Figure S7

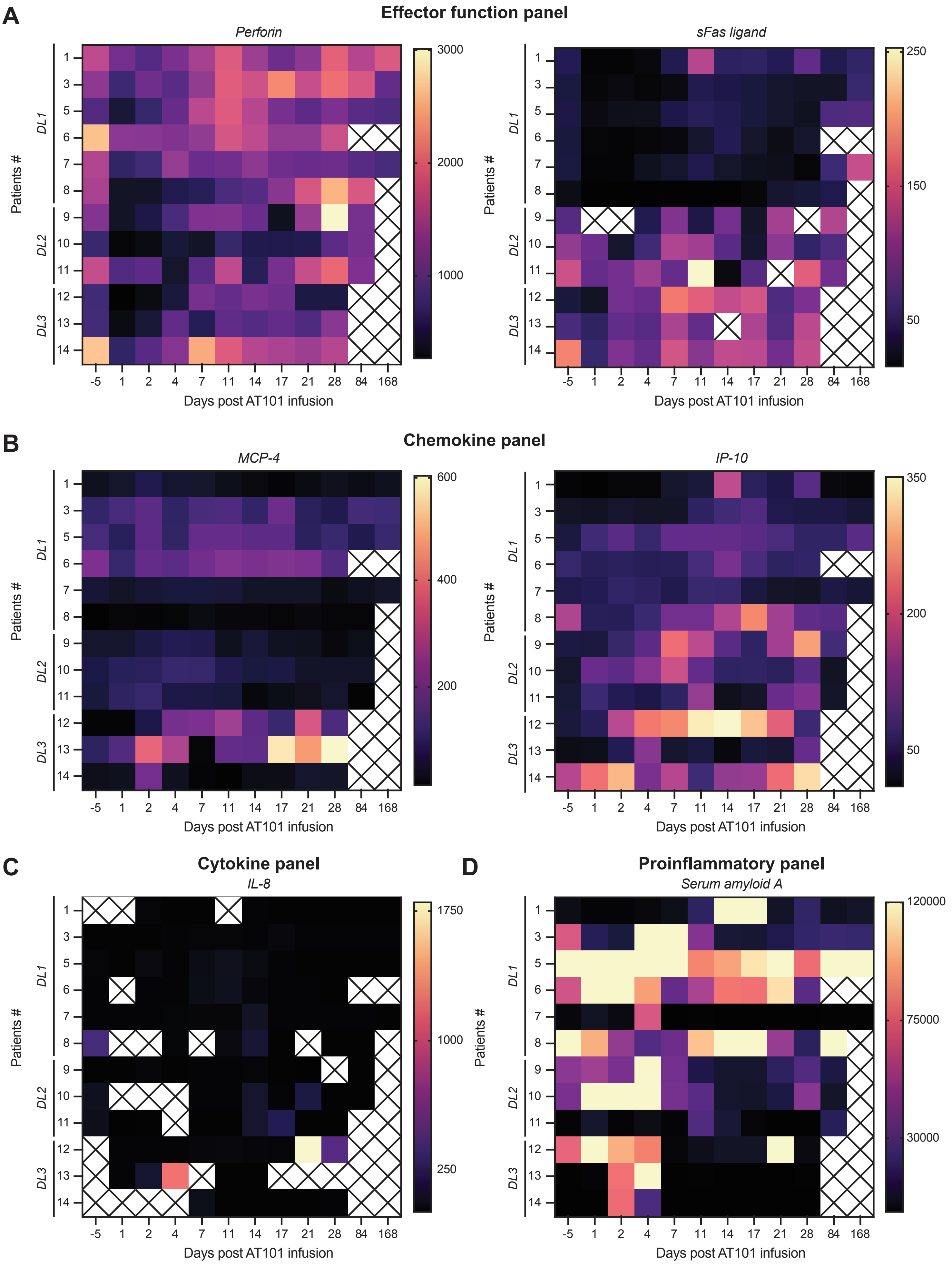
A
Effector function panel
Perforin
sFas ligand
B
Chemokine panel
MCP-4
IP-10
C
Cytokine panel
IL-8
D
Proinflammatory panel
Serum amyloid A
Patients #
DL1
DL2
DL3
1
3
5
6
7
8
9
10
11
12
13
14
Days post AT101 infusion
-5
1
2
4
7
11
14
17
21
28
84
168
3000
2000
1000
250
150
50
600
400
200
350
200
50
1750
1000
250
120000
75000
30000
